# AOP Report: Uncoupling of Oxidative Phosphorylation Leading to Growth Inhibition via Decreased Cell Proliferation

**DOI:** 10.1002/etc.5197

**Published:** 2021-10-14

**Authors:** You Song, Daniel L. Villeneuve

**Affiliations:** a Norwegian Institute for Water Research, Oslo, Norway; b Great Lakes Toxicology and Ecology Division, US Environmental Protection Agency, Duluth, Minnesota, USA

**Keywords:** Adverse outcome pathway, Hazard/risk assessment, Toxicity mechanisms

## Abstract

This report describes a novel adverse outcome pathway (AOP) on uncoupling of oxidative phosphorylation (OXPHOS) leading to growth inhibition via decreased adenosine triphosphate (ATP) pool and cell proliferation (AOPWiki, AOP263). Oxidative phosphorylation is a major metabolic process that produces the primary form of energy (ATP) supporting various biological functions. Uncoupling of OXPHOS is a widely recognized mode of action of many chemicals and is known to affect growth via different biological processes. Capturing these events in an AOP can greatly facilitate mechanistic understanding and hazard assessment of OXPHOS uncouplers and growth regulators in eukaryotes. The four proposed key events in this AOP are intentionally generalized to cover a wide range of organisms and stressors. Three out of four events can be measured using in vitro high-throughput bioassays, whereas for most organisms, growth inhibition can also be measured in a high-throughput format using standard in vivo toxicity test protocols. The key events and key event relationships in this AOP are further assessed for weight of evidence using evolved Bradford-Hill considerations. The overall confidence levels range from moderate to high with only a few uncertainties and inconsistencies. The chemical applicability domain of the AOP mainly contains protonophores uncouplers, which can be predicated using the quantitative structure-activity relationship (QSAR) approach and validated using in vitro high-throughput bioassays. The biological domain of the AOP basically covers all eukaryotes. The AOP described in this report is part of a larger AOP network linking uncoupling of OXPHOS to growth inhibition, and is considered highly relevant and applicable to both human health and ecological risk assessments.

## INTRODUCTION AND BACKGROUND

**FIGURE 1:**
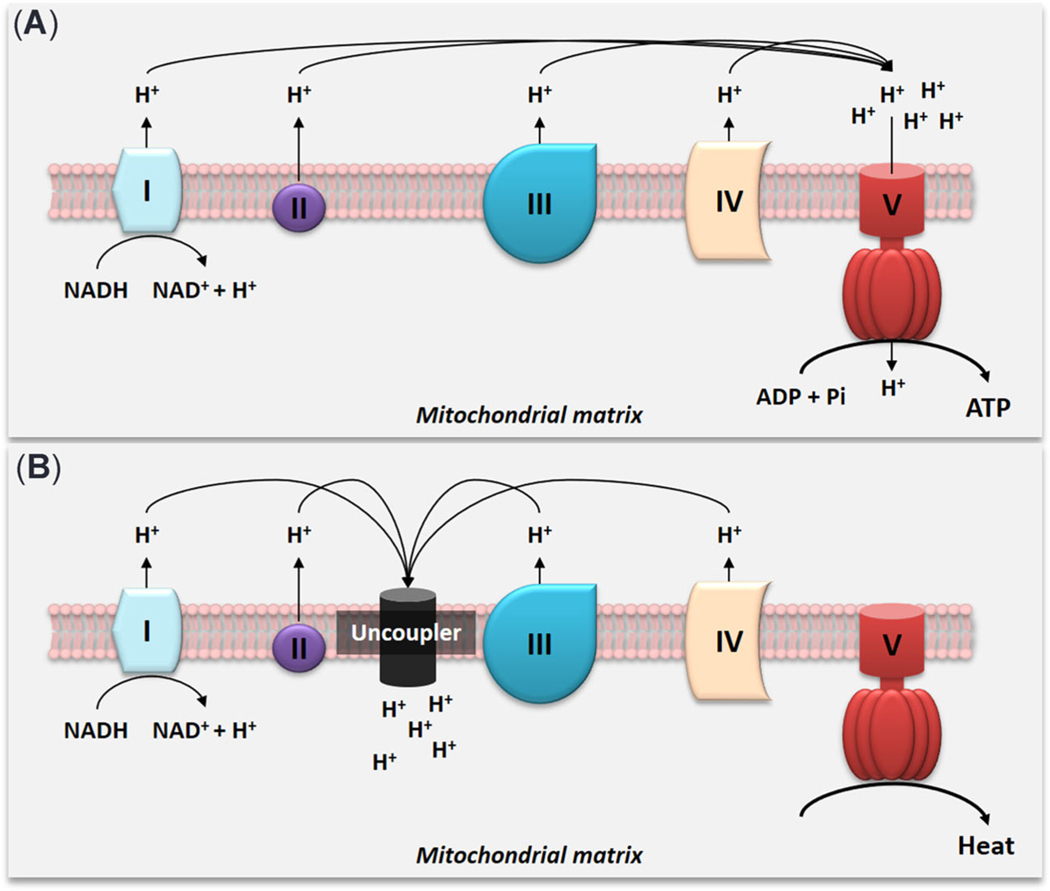
Coupling (**A**) and uncoupling (**B**) of oxidative phosphorylation. NADH = reduced nicotinamide adenine dinucleotide; ADP = adenosine diphosphate; Pi = inorganic phosphate; ATP = adenosine triphosphate.

Oxidative phosphorylation (OXPHOS) is a major metabolic process that produces adenosine triphosphate (ATP), a primary form of energy supporting various biological functions. Oxidative phosphorylation mainly takes place in mitochondria, where a chain of redox reactions using reduced nicotinamide adenine dinucleotide (NADH) or flavin adenine dinucleotide (FADH_2_) as a substrate is coupled with phosphorylation of adenosine diphosphate (ADP) to produce ATP (Liberman et al., 1969). The redox reactions are mediated by four protein complexes (Complexes I–IV) in an electron transport chain to generate a proton motive force (Δp) across the inner mitochondrial membrane. The proton motive force is the main driver of ATP synthesis by F0–F1 ATP synthase (Complex V; Figure 1A). According to the chemiosmotic theory (Mitchell, 1961, 1966), the proton motive force consists of an electrical mitochondrial membrane potential (ΔΨ) and a proton gradient across the inner mitochondrial membrane (ΔpH). The mitochondrial membrane potential normally contributes to 80 to 85% of the proton motive force (reviewed in Dzbek & Korzeniewski, 2008) and has been widely used as an indicator of proton motive force. The OXPHOS machinery is considered the most efficient ATP synthetic process in eukaryotes.

Environmental stressors can affect OXPHOS through direct or indirect mechanisms of action (Hanstein, 1976). Chemicals that can dissipate proton motive force and uncouple OXPHOS are commonly referred to as uncouplers (Kadenbach, 2003). The uncoupling effect was discovered more than 70 years ago: the weight loss agent 2,4-dinitrophenol (DNP) was found to uncouple mitochondrial oxidation and phosphorylation in rabbit kidney homogenates (Loomis & Lipmann, 1948). During a chemical uncoupling action, an uncoupler binds to a proton in the inter membrane space, transports the proton across the inner mitochondrial membrane, releases the proton into the other (matrix) side of the membrane, and diffuses back to the intermembrane space. This process is repeated until the transmembrane proton motive force is fully dissipated (Terada, 1990). In general, uncouplers only diminish the efficiency of ATP synthesis without directly affecting the functions and activities of the complexes in the electron transport chain (Terada, 1990). Instead of producing ATP, heat is generated under an uncoupling situation (Figure 1B).

It is widely accepted that typical uncouplers have three common structural characteristics: bulky hydrophobic moiety, an acid dissociable group, and a strong electron-withdrawing group (Terada, 1990). Most uncouplers are protonophores with strong abilities to diffuse across the membranes (Kessler et al., 1976). Weak acids, such as phenols, salicylanilides, and aromatic amines are well-known protonophoric uncouplers (McLaughlin & Dilger, 1980). Classical uncouplers, such as 2,4-DNP, carbonyl cyanide-p-trifluoromethoxyphenyl hydrazone, pentachlorophenol, and 3,5-di-t-butyl-4-hydroxybenzylidinemalononitrile have been widely used as positive controls in (eco)toxicological tests (see Schultz et al., 2002), whereas the hazards of “new” uncouplers, such as triclosan, emodin, and metabolites of polybrominated diphenyl ethers (PBDEs) are under extensive assessments (see Sugiyama et al., 2019). Predictive approaches such as quantitative structure–activity relationships (QSARs; see Dreier et al., 2019a), biophysical models (see Ebert & Goss, 0014), and classification approaches such as toxicogenomics (see Hawliczek-Ignarski et al., 2017) have been developed to identify new organic uncouplers. There are also endogenous uncouplers for regulating energy homeostasis and body temperature, such as uncoupling proteins (reviewed in Rousset et al., 2004) and fatty acids (reviewed in Kadenbach, 2003). Mild uncoupling can normally enhance the oxygen consumption rate, electron transport chain activity, and/or other ATP synthetic processes (e.g., glycolysis) as compensatory mechanisms to restore ATP supply (Desquiret et al., 2006; Weinbach, 1957).

Growth is a regulatory relevant chronic toxicity endpoint and has been included in multiple Organisation for Economic Cooperation and Development (OECD) test guidelines, as well as in adverse outcome pathways (AOPs; Groh et al., 2015). Tissue, organ, and organismal growth are mainly achieved through cell proliferation, a highly ATP-dependent process representative of the combined outcome of cell growth (increase in volume), cell division (increase in number), and cell death (decrease in number). Abnormal cell proliferation (e.g., tumor growth) can be attributed to many causes, with mitochondrial energetic dysfunction being one of the most intensively investigated. Because cellular ATP is a determinant factor of various signaling and metabolic pathways responsible for cell cycle regulation and major cellular component biosynthesis (Bonora et al., 2012), cellular energy status and proliferation are tightly coupled. In vertebrates, it has been suggested that the adenosine monophosphate (AMP)-activated protein kinase (AMPK) signaling pathway is responsible for sensing cellular energy status and regulating cell proliferation rate via a series of downstream mechanisms (reviewed in Hardie, 0018), including the widely studied target of rapamycin (TOR) pathway as a master regulator of programmed cell death, protein synthesis, and lipid metabolism (reviewed in Wullschleger et al., 2006). The exact functions of these pathways, however, have not been fully elucidated for invertebrates and primary producers.

On the basis of the current knowledge, it is evident that a series of events mediated by OXPHOS uncouplers can be causally connected in an AOP network. In the present study, we elaborate on one of the AOPs in the network linking uncoupling of OXPHOS, ATP depletion, impaired cell proliferation, and growth inhibition.

The motivation for developing this AOP originates from frequent observations of reduced mitochondrial membrane potential and ATP synthesis as common effects of environmental stressors (e.g., chemicals, radiation, heat stress). Capturing these events in an AOP can greatly facilitate mechanistic understanding and hazard assessment of relevant stressors. Several recent publications have also pointed out the need for including mitochondrial dysfunctions in the AOP framework (see Bolser et al., 2018; Dreier et al., 2019b). Although a number of AOPs in the AOP-Wiki (Society for the Advancement of Adverse Outcome Pathways [SAAOP], 2020) have considered key events related to mitochondrial dysfunction, only four AOPs (AOP-Wiki: AOPs 3, 256, 273, and 335) directly address mitochondrial toxicity by using relevant molecular initiating events (MIEs), such as inhibition of electron transport chain complexes and damage to mitochondrial DNA. Our RiskAOP project (Norwegian Institute for Water Research, 2020) therefore aimed to better integrate mitochondrial endpoints in the AOP framework and develop quantitative AOPs to facilitate risk assessment of mitochondrial toxicants.

With the motivations and project support in place, a set of conceptual AOPs linking uncoupling of OXPHOS to growth inhibition was assembled and submitted to the AOP-Wiki (AOPs 263–268). These AOPs are considered highly relevant and applicable to both human health and ecological risk assessments. Whereas the AOP we present (AOP 263) represents an important part of a broader network, it is understood that other, intermediate, key events may also contribute to growth inhibition. For example, generation of mitochondrial reactive oxygen species (mtROS) and subsequent oxidative stress due to abnormal redox reactions, impaired lipid metabolism due to loss of energy homeostasis, and programmed cell death due to oxidative damage can all occur as a result of OXPHOS uncoupling. The proposed AOP network therefore considers various consequences of mitochondrial uncoupling as key events such as mtROS formation, DNA damage, protein oxidation, lipid peroxidation, and cell death. Detailed descriptions of the likely concurrent key events and their relationships to OXPHOS uncoupling and/or growth inhibition will be addressed elsewhere.

It should also be noted that growth inhibition is likely not the only adverse outcome following uncoupling of OXPHOS. Other types of uncoupler-mediated adverse effects, such as teratogenesis, reduced fertility, neurodegeneration, and cardiac diseases may also be considered when one is assembling information into a larger AOP network for mitochondrial dysfunction. Although the present study focuses on growth inhibition, that is just one of several potential adverse outcomes that may be linked to uncoupling of OXPHOS. Moreover, it is biologically plausible that population decline is a potential higher level adverse outcome linked to growth inhibition, but the relationship warrants further development for empirical support and is not included in the AOP we describe in the present study. *Environ Toxicol Chem* 2021;00:1–9.

## AOP DESCRIPTION

**FIGURE 2:**
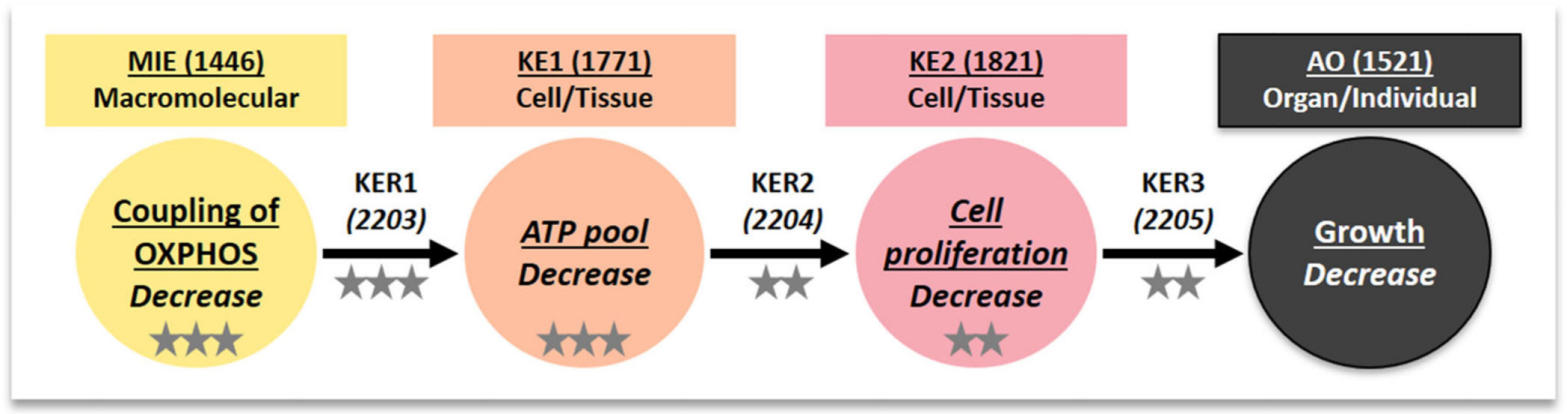
Graphical representation of the adverse outcome pathway (AOP). MIE = molecular initiating event; KE = key event; AO = adverse outcome; KER = key event relationship; OXPHOS = oxidative phosphorylation; ATP = adenosine triphosphate. The numbers in parentheses indicate the event ID (KE) or relationship ID (KER) in the AOP-Wiki. The overall confidence level is indicated by the number of stars (3, high; 2, moderate; 1, low).

The proposed AOP (Figure 2 and Textbox 1) links decreased coupling (uncoupling) of OXPHOS (MIE) to decreased growth (adverse outcome) via two key events, decreased ATP pool (KE1) and decreased cell proliferation (KE2). Briefly, partitioning of protonophores (uncouplers) into the inner mitochondrial membrane is known to uncouple OXPHOS, leading to dissipation of proton motive force and subsequent reduction in ATP synthesis. Because many biological processes require ATP, reduced ATP synthesis can rapidly lead to depletion of the cellular ATP pool. Lack of sufficient energy supply can hamper cell proliferation and ultimately cause growth inhibition.

The four proposed key events were intentionally generalized to cover a wide range of organisms and stressors. The MIE “decrease, coupling of OXPHOS” is a lumped term representative of the main outcome of three initial actions of an uncoupler: binding of protons in the intermembrane space, transportation of protons across the inner mitochondrial membrane (uncoupling action), and dissipation of proton motive force. The first two actions are considered difficult to measure, whereas the third can be proportionally indicated by mitochondrial membrane potential, proton leak, and/or oxygen consumption rate. The three intermediate events are therefore considered as Key Event Components (Ives et al., 2017) to support the MIE in the AOP-Wiki. It should be noted that “dissipation of proton motive force” is an important event that is relevant to many other AOPs and has a great potential to be considered as an independent key event in the future with the evolvement of knowledge and analytical technology. Uncoupling of OXPHOS is used in the name of the AOP because it is a widely used term to describe a specific mechanism of action of chemicals and thus is familiar to researchers and regulators.

The first key event, “decrease, ATP pool,” represents the combined outcome of reduced mitochondrial ATP biosynthesis and increased ATP depletion due to various ATP-consuming processes. The available ATP pool can be easily quantified. In contrast, the ATP synthetic process is highly dynamic and thus more difficult to measure, even though it mechanistically serves as the bridge between uncoupling and decreased total ATP. The second key event, “decrease, cell proliferation,” is the outcome of a dynamic process integrating cell division, cell growth, and cell death. These processes taken together determine the rate of tissue and organismal growth. It is recognized that multiple biological events exist between KE1 and KE2 to regulate both cellular energy homeostasis and proliferation, with the AMPK–TOR cascade being key. However, the underlying pathways linking ATP abundance to cell proliferation have a high level of cross-talk with other pathways, and their responses may be difficult to measure and/or predict. In addition, there is a lack of understanding and supporting evidence for the more detailed molecular and biochemical events underpinning energetic control of cell proliferation in invertebrates and plants that are intended to be covered by this AOP. Thus, cell proliferation itself was viewed as the next practical check-point to include in the causal chain of biological events. The MIE, KE1, and KE2 can be measured using in vitro high-throughput screening bioassays (Textbox 2). The adverse outcome “decrease, growth” addresses a chronic endpoint of regulatory concern and can be easily quantified by measuring the weight or length of a tissue/organ (large organism) or individual organism (small organism) in standard in vivo (eco)toxicity tests. For unicellular organisms such as algae, population growth (total cell number or volume) can be considered as the adverse outcome.

## SUMMARY OF SCIENTIFIC EVIDENCE ASSESSMENT

As a whole, weight of evidence supporting an AOP is intended to provide scientific support that the sequence of key events outlined is causally related to one another. Evidence supporting causality was evaluated based on the evolved Bradford–Hill considerations (Becker et al., 2015). The strength of scientific evidence was subjectively rated as “high,” “moderate,” or “low,” according to the instructions in the OECD’s Guidance Document for Developing and Assessing AOPs (OECD, 2018).

### Essentiality of the key events

Evidence for essentiality documents that in the presence of a stressor that activates an AOP, then cessation of exposure, knockout, inhibition, blockage, or modulation of an upstream key event can prevent the occurrence of a downstream key event, or lead to recovery of normal biological processes (OECD, 2018). Consequently, specifically designed (eco)toxicological studies with known uncouplers as the stressors, such as exposure–recovery tests, knockout–rescue tests, and blockage–continuation tests, as well as modulation–response tests, were used to evaluate the essentiality of key events. The essentiality of the MIE (*Event 1446: Decrease, Coupling of OXPHOS*) is considered high, because direct evidence was collected from multiple specifically designed studies showing simultaneous recovery of OXPHOS coupling (as indicated by the membrane potential) and ATP pool due to removal of the uncouplers (see Weisová et al., 2012), or addition of OXPHOS recoupling agents (see Sibille et al., 1995). The essentiality of KE1 (*Event 1771: Decrease, ATP pool*) is considered high, as supported by direct evidence from a typical inhibition–rescue study showing restimulation of cell proliferation by ATP (Wang et al., 2017) and multiple lines of indirect evidence showing strong positive correlations between ATP and cell proliferation (see Sweet & Singh, 1999). The essentiality of KE2 (*Event 1821: Decrease, Cell proliferation*) is considered moderate, due to a lack of specifically designed studies to provide direct evidence. Several studies, however, have shown positive correlations between cell proliferation and growth (see Figarola et al., 2015), thus providing indirect evidence to support the essentiality of this key event.

### Biological plausibility of the key event relationships

Biological plausibility describes the known structural or functional relationships between an upstream key event and a downstream key event under normal conditions or unperturbed biology (OECD, 2018). Such fundamental biological understanding provides the ability to infer or hypothesize the likely consequences of a biological perturbation elicited by a stressor. The biological plausibility of key event relationship (KER)1 (*Relationship 2203: Decrease, Coupling of OXPHOS leads to Decrease, ATP pool*) is considered high. The cellular ATP pool represents a balance between ATP synthesis and ATP consumption (Bonora et al., 2012). Reduced ATP production or elevated demand for energy can rapidly cause ATP depletion. Among all ATP synthetic pathways, mitochondrial OXPHOS supplies the majority of the cellular ATP in eukaryotes (SchmidtRohr, 2020). Uncoupling of OXPHOS can reduce the ATP pool by affecting the ATP synthetic efficiency. For KER2 (*Relationship 2204: Decrease, ATP pool leads to Decrease, Cell proliferation*), the biological plausibility is considered high. Because ATP is essential for cell division and macromolecule biosynthesis (Bonora et al., 2012), cell proliferation is highly dependent on available ATP. Depletion of the ATP pool can therefore lead to reduced cell proliferation. High biological plausibility is also found for KER3 (*Relationship 2205: Decrease, Cell proliferation leads to Decrease, Growth*). Because eukaryotic growth is mainly achieved by increasing the total cell number and volume (Conlon & Raff, 1999), reduced cell proliferation (division and growth) is thus considered a main cause of growth inhibition.

### Empirical support for the key event relationships

The anticipated patterns of response that support a causal relationship between an upstream key event (Event A) and a downstream key event (Event B) are: (1) Event A is impacted at a concentration/dose of stressor that is less than or equal to that which impacts Event B (dose–response concordance); (2) Event A occurs before Event B is observed (temporal concordance); and (3) Event A is observed in a proportion of the sample population equal to or greater than that in which Event B is observed (incidence concordance; OECD, 2018). Consequently, studies reporting measurements of at least two key events in the same biological system after exposure to an uncoupler, with a minimum of one common dose and/or time point(s) were considered in the assessment of empirical evidence (Supporting Information, Table S1).

The empirical support of KER1 (*Relationship 2203: Decrease*, *Coupling of OXPHOS leas to Decrease, ATP pool*) is considered high, because good incidence (see Sweet & Singh, 1999), temporal (see Bestman et al., 2015), and dose concordance (see Weatherly et al., 2018) have been shown by multiple studies, with a limited number of inconsistencies (Supporting Information, Table S1). Moderate empirical supports were found for KER2 (*Relationship 2204: Decrease, ATP pool leads to Decrease, Cell proliferation*). Temporal and incidence concordance have been shown by several studies (see Sithara et al., 2017), with one study also reporting an inconsistent observation (Kuruvilla et al., 2003). Due to a lack of in vivo or ex vivo studies covering both key events in the same experiment, low empirical support is considered for KER3 (Relationship 2205: *Decrease, Cell proliferation leads to Decrease, Growth*). There is, however, one study partially showing temporal concordance between reduced cell proliferation and zebrafish embryo growth (Bestman et al., 2015).

### Uncertainties, inconsistencies, and critical gaps

There are several uncertainties related to this AOP. First, although uncoupling of OXPHOS has been extensively investigated, no study has simultaneously included all key events in the same analysis. Supporting evidence was summarized based on propagated information collected from multiple doses/concentrations, time points, cell types, or species. This raises a concern about the overall relationship of the key events in this AOP. Second, some evidence was generated using tumor cells. There is uncertainty whether these cells respond to uncouplers in a similar manner as normal cells. There can also be large tissue-specific effects, and not every cell type is equally susceptible (see Bestman et al., 2015). Third, full (i.e., more than five test doses/concentrations) dose–response analysis is lacking in most of the studies, leading to uncertainty about the concordance across multiple doses as well as difficulties in deriving threshold values of perturbation such as point of departure. Fourth, a few studies did not perform statistical analysis or clearly report the effect doses/concentrations. The AOP developers were only able to estimate approximate values from the figures in their reports. Fifth, like many other AOPs, the uncertainty increases with an increase in the level of biological organization. For example, growth inhibition at the apical level is likely not only attributed to impaired cell proliferation, but also to other effects. This requires further development of the associated AOP network in the context of regulatory applications. Sixth, although the AOP is proposed for animals and plants in general, there is a significant lack of supporting data from invertebrates and plants. This calls for more follow-up studies using nonvertebrate models such as insects, crustaceans, nematodes, and mollusks. Seventh, only a limited number of studies have considered glycolysis as a compensatory mechanism to uncoupling of OXPHOS. The ATP pool may show marginal changes due to activation of alternative ATP synthetic pathways such as glycolysis (Marroquin et al., 2007). It should also be noted that the regulation of compensatory mechanisms may also require additional consumption of energy. Therefore, the observed change in the ATP pool is expected to be influenced by multiple upstream biological processes.

Some inconsistencies were also identified concerning the empirical support of the KERs, such as an incidence of increased ATP after exposure to an uncoupler (Kuruvilla et al., 2003), and more sensitive changes in the adverse outcome compared with an earlier key event (Bestman et al., 2015; Xie et al., 2018). These inconsistencies are likely attributed to a combination of nonoptimal sampling time points for analysis and potential compensatory mechanisms, such as glycolysis during chronic low-dose exposures (Jose et al., 2011), which need to be further considered for the quantitative aspect of the AOP.

### Quantitative understanding

Quantitative understanding describes whether the magnitude or probability of a downstream key event can be mathematically predicted by the magnitude or probability of an upstream key event with known uncertainties (OECD, 2018). Studies that report the quantitative response–response relationships of at least two key events, with or without an uncoupler exposure, were considered to support quantitative understanding of the KERs.

The quantitative understanding of KER1 (*Relationship 2203: Decrease, Coupling of OXPHOS leads to Decrease, ATP pool*) is considered high, because multiple mathematical models describing the quantitative relationships between uncoupling of OXPHOS and ATP synthesis in vertebrates have been developed (see Beard, 2005). There are also case-specific studies reporting the quantitative relationship between ATP synthesis and ATP consumption in vertebrates (see Matsuda et al., 2020). In invertebrates, a regression-based quantitative response–response relationship between uncoupling of OXPHOS and ATP depletion has been proposed for the crustacean *Daphnia magna* under UVB stress (Song et al., 2020).

The quantitative understanding of KER2 (*Relationship 2204: Decrease, ATP pool leads to Decrease, Cell proliferation*) was considered moderate. The quantitative relationships between total ATP and cell proliferation have been extensively investigated (Crouch et al., 1993). In general, a monotonic positive relationship can be assumed for the two events, although the actual quantitative relationship can vary across biological systems (e.g., cell types and species). It has also been suggested that a threshold of ATP depletion (85–90% reduction compared with normal status) may exist to determine whether proliferation arrest (less than 85–90%) or cell death (more than 85–90%) will be triggered in mammals (Nieminen et al., 1994).

The quantitative understanding of KER3 (*Relationship 2205: Decrease, Cell proliferation leads to Decrease, Growth*) is also considered moderate. Multiple mathematical models describing the quantitative relationships between cell proliferation and tissue growth exist for both animals (Binder et al., 2008; Stadnicka-Michalak et al., 2015) and plants (Mosca et al., 2018). There are also numerous models that are specifically developed for predicting tumor growth based on the proliferation rate (Jarrett et al., 2018). However, there is currently a lack of quantitative models to link cell proliferation and individual growth in the presence of uncouplers.

### Environmental relevance

The AOP is considered environmentally relevant because it addresses a chronic sublethal toxicity endpoint (growth) that is common for almost all living organisms (including humans). Although the reported effect concentrations/doses to trigger the key events are relatively high (i.e., μM or higher), these were mostly derived from acute tests. It has been suggested by Raimondo and colleagues (2007) that the median acute-to-chronic ratio for all documented chemicals affecting growth in aquatic invertebrates and fish is 10.2 (based on 188 studies), or 6.2 for OXPHOS uncouplers leading to various types of adverse effects in different organisms (based on 26 studies). It is plausible that effect concentrations that trigger the AOP in a chronic test fall in the range of environmentally realistic levels. For example, the measured environmental concentrations of triclosan ranged from 1.4 to 40,000 ng/L in surface waters and from 20 to 86,161 ng/L in wastewater influent (Dhillon et al., 2015), whereas the reported median effect concentration to cause chronic growth inhibition in algae was lower (1400–19,000 ng/L; Orvos et al., 2002).

### Domains of applicability

The empirical evidence (Supporting Information, Table S1) suggests that the AOP is potentially applicable to zebrafish (*Danio rerio*), human (*Homo sapiens*), rat (*Rattus norvegicus*), mouse (*Mus musculus*), and common duckweed (*Lemna minor*). Because the key events are highly generalized and linked to common biological processes in eukaryotes, the taxonomic applicability domain can likely cover most animals, plants, and algae. The AOP is in general considered sex-unspecific, because the key events are equally relevant for both males and females. The response patterns of the key events, however, can vary dramatically between males and females (see Schwetz et al., 1978). Although all life stages are potentially relevant for this AOP, embryos and juveniles are considered particularly susceptible to growth inhibitors. One of the seminal functions of this AOP could be guiding direct testing to define the boundaries of its applicability domains.

## POTENTIAL APPLICATIONS

A variety of in silico and in vitro methods have been used to identify chemicals likely to act as uncouplers of oxidative phosphorylation (see Xia et al., 2018; Dreier et al., 2019a; Supporting Information, Table S2, for a list of potential uncouplers identified by the Tox21 quantitative high-throughput screening [qHTS] program). The weight of evidence assembled part of this AOP identifies these compounds as potential growth inhibitors, thus flagging them as chemicals of concern. It identifies three key event measurements, most of which can be measured in high-throughput, which can be used to confirm activity along this AOP and characterize effective doses. Thus, in the near term the AOP is most applicable to hazard identification and subsequent tiered testing or integrated approaches to testing and assessment. However, with the moderate to high quantitative understanding available for this pathway, quantitative application to higher tier assessments may be possible. Additional testing of quantitative predictions is needed before confidence in those predictions can be established. Given the diversity of chemical structures that may function as uncouplers, quantitative understanding of the AOP will need to be complemented with effective toxicokinetic models to reasonably predict the severity of uncoupling at the level of the target organ or organism. Nonetheless, overall the present AOP has the potential to: (1) link existing in vitro and in silico data to a regulatory relevant endpoint (i.e., growth); (2) improve safety assessment of mitochondrial uncouplers by providing a tiered testing strategy; and (3) cover organisms from multiple trophic levels.

## Supplementary Material

SI1

SI2

## Data Availability

Data, associated metadata, and calculation tools are available from the corresponding author (you.song@niva.no).

## TEXTBOX 1: AOP ID

- Formal AOP title: Uncoupling of oxidative phosphorylation leading to growth inhibition via decreased cell proliferation
- AOP authors: You Song and Daniel L. Villeneuve
- AOP number: 263
- Development status: EAGMST under review
- OECD work plan project number: 1.92
- List of key events:
  - MIE: Decrease, Coupling of oxidative phosphor-ylation (Event ID 1446)
  - KE1: Decrease, Adenosine triphosphate pool (Event ID 1771)
  - KE2: Decrease, Cell proliferation (Event ID 1821)
  - Adverse outcome: Decrease, Growth (Event ID 1521)

## TEXTBOX 2: List of assays associated with the AOP

- MIE: Uncoupling of oxidative phosphorylation can be indicated by a decrease in mitochondrial membrane potential, an increase in proton leak, and/or an increase in oxygen consumption rate. Widely accepted high-throughput methods for determination of mitochondrial membrane potential in isolated mitochondria, cells, or tissue are mainly based on commercially available fluorescent probes, such as tetramethylrhodamine methyl ester (TMRM), tetramethylrhodamine ethyl ester (TMRE), and JC-1. Oxygen consumption rate can be measured using a high-resolution respirometer or equivalent such as a Seahorse XF Analyzer. It is highly recommended to combine different types of measurements to verify whether a chemical has an uncoupling mode of action. Standardized ToxCast high-throughput screening bioassays, such as “APR_HepG2_Mito-MembPot,” “APR_Hepat_MitoFxnI,” and “APR_Mitochondrial_membrane_potential”, and the Tox21 high-throughput screening assay “tox21-mitotox-p1” can be used to measure this MIE.
- KE1: The ATP pool in cells or tissues sampled can be measured using commercially available ATP bioluminescence assay kits. Standardized ToxCast high-throughput screening bioassays, such as “NCCT_HEK293T_CellTiterGLO” and “NIS_HEK293T_CTG_Cytotoxicity” can be used to measure this key event.
- KE2: Cell proliferation can be measured using commercially available kits such as 5-bromo-2′-deoxyuri dine (BrdU) assay and 5-ethynyl-2´-deoxyuridine (EdU) assays. Standardized ToxCast high-throughput screening bioassays such as “BSK_3C_Proliferation,” “BSK_CASM3C_Proliferation,” and “BSK_SAg_Proliferation” can be used to measure this key event.
- Adverse outcome: Growth can be indicated by measuring the weight and/or length of an organ or a whole organism in animals and plants. For algae, total cell number or total cell volume can be used to indicate growth. Standardized OECD test guidelines, such as 206, 208, 201, 210, 211, 212, 215, 221, 228, and 241 can be used to measure this adverse outcome in different species.
