# Supplementary material for: AOP Report: Uncoupling of Oxidative Phosphorylation Leading to Growth Inhibition via Decreased Cell Proliferation
Source: Environ Toxicol Chem. Author manuscript; Available in PMC 2023 Nov 2. (PMC10620627; doi:10.1002/etc.5197)
Supplement: SI2 [file NIHMS1748635-supplement-SI2.pdf]

AOP ID and Title:

AOP 263: Uncoupling of oxidative phosphorylation leading to growth inhibition via decreased cell proliferation
Short Title: Uncoupling of OXPHOS leading to growth inhibition 1

Graphical Representation

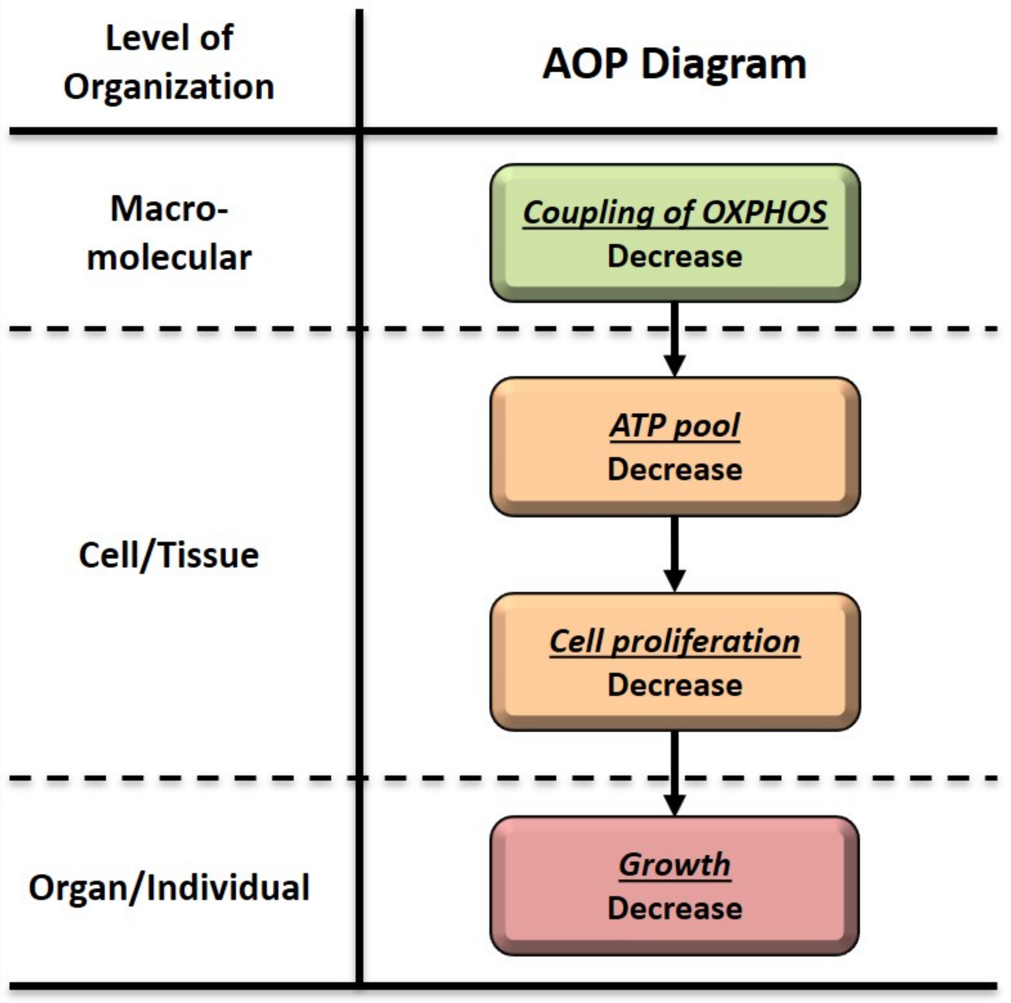

Authors

You Song[a] and **Daniel L. Villeneuve**[b]

[a] Norwegian Institute for Water Research (NIVA), Gaustadalléen 21, NO-0349 Oslo, Norway

[b] U.S. Environmental Protection Agency, Great Lakes Toxicology and Ecology Division, Duluth, Minnesota 55804, USA

Acknowledgement

This project was funded by the Research Council of Norway (RCN), grant no. 301397 “RiskAOP - Quantitative Adverse Outcome Pathway assisted risk assessment of mitochondrial toxicants” (https://www.niva.no/en/projectweb/riskaop), and supported by the NIVA Computational Toxicology Program, NCTP (www.niva.no/nctp).

Status

Author status	OECD status	OECD project	SAAOP status
Open for comment. Do not cite	Under Development	1.92	Included in OECD Work Plan

Abstract

Uncoupling of oxidative phosphorylation (OXPHOS) is a well-known mechanism of action of many chemicals. Mitochondrial uncoupler-mediated energetic dysfunction is known to affect growth, a critical process in most organisms and a chronic toxicity endpoint included in many OECD test guidelines. This adverse outcome pathway (AOP) causally links uncoupling of OXPHOS to growth inhibition, through ATP depletion and reduced cell proliferation as the intermediate key events (KEs), with strong weight of evidence support. The AOP is generalized to reflect its expected applicability to a broad range of taxa, ranging from microalga to human. Three out of four KEs included can be quantified using high-throughput methods, making this AOP particularly useful for screening, prioritization and hazard assessment of mitochondrial uncouplers as potential growth inhibiting chemicals. This AOP is of high regulatory relevance, as it is considered applicable to both human health and ecological risk assessments. The AOP also forms the core of a larger AOP network addressing uncoupling of OXPHOS mediated growth inhibition (AOP 263-268).

Background

The mitochondrial OXPHOS machinery is a key physiological process responsible for producing the primary cellular energy, adenosine triphosphate (ATP). During OXPHOS, a series of redox reactions (oxidation) are mediated by protein complexes in an electron transport chain to create a protonmotive force (PMF) across the inner mitochondrial membrane (Liberman 1969). The PMF acts as a driving force of ATP synthesis through phosphorylation of adenosine diphosphate (ADP). Mitochondrial oxidation and phosphorylation are coupled to ensure continuous ATP supply for various physiological processes. A number of chemicals can bind to the inner mitochondrial membrane and dissipate the PMF, thus leading to uncoupling of OXPHOS and reduction in ATP synthetic efficiency. Classical “uncouplers” are normally protonophores with major characteristics of bulky hydrophobic moiety, an acid dissociable group and a strong electron-withdrawing group (Terada 1990). With the rapid development of in silico (Russom 1997; Schultz 1997; Naven 2012; Dreier 2019; Troger 2020) and in vitro (Escher 2002; Attene-Ramos 2013; Attene-Ramos 2015; Xia 2018) approaches, more and more uncouplers have been identified. However, their hazards to biota remain to be assessed. Uncoupling of OXPHOS can affect many ATP-dependent biological functions. In particular, cell proliferation as a major process to achieve organismal growth is positively correlated with the cellular ATP level and highly susceptible to energy depletion (Ramaiah 1964; Bonora 2012). Therefore, a link between uncoupling of OXPHOS and growth inhibition can be established with ATP depletion and reduced cell proliferation as the intermediate steps.

Summary of the AOP

Events

Molecular Initiating Events (MIE), Key Events (KE), Adverse Outcomes (AO)

Sequence	Type	Event ID	Title	Short name
	MIE	1446	Decrease, Coupling of oxidative phosphorylation	Decrease, Coupling of OXPHOS
	KE	1771	Decrease, Adenosine triphosphate pool	Decrease, ATP pool
	KE	1821	Decrease, Cell proliferation	Decrease, Cell proliferation
	AO	1521	Decrease, Growth	Decrease, Growth

Key Event Relationships

Upstream Event	Relationship Type	Downstream Event	Evidence	Quantitative Understanding
Decrease, Coupling of oxidative phosphorylation	adjacent	Decrease, Adenosine triphosphate pool	High	High
Decrease, Adenosine triphosphate pool	adjacent	Decrease, Cell proliferation	Moderate	Moderate
Decrease, Cell proliferation	adjacent	Decrease, Growth	Moderate	Moderate

Stressors

Name	Evidence
2,4-Dinitrophenol	High
Pentachlorophenol	High
Carbonyl cyanide-p-trifluoromethoxyphenylhydrazone	High
Carbonyl cyanide m-chlorophenyl hydrazone	High

Name	Evidence
Triclosan	High
Dinoseb	Moderate
3,5-Dichlorophenol	Moderate
Emodin	High
Arsenite	High

Overall Assessment of the AOP

The weight of evidence (WoE) assessment of the AOP was conducted based on the evolved Bradford-Hill considerations (Becker 2015) and according to the criteria in OECD's Guidance Document for Developing and Assessing AOPs (OECD 2018). In terms of evidence for the essentiality of the key events, the MIE (Event 1446) and KE1 (Event 1771) were scored as high, whereas KE2 (Event 1821) was scored as moderate due to a lack of solid evidence to support its essentiality. The overall WoE of KER1 (Relationship 2203) is considered high, as strong biological plausibility, empirical evidence and fairly good quantitative understanding were evidenced from multiple studies. The overall WoE of KER2 (Relationship 2204) is considered moderate, due to high biological plausibility, acceptable empirical concordance and some biological understanding. The overall WoE of KER3 (Relationship 2205) is scored as moderate, mainly due to biological plausibility, but there is presently a lack of empirical evidence and quantitative understanding to further support causality. The AOP is considered applicable to a wide range of species as well as a broad domain of chemicals. The rationales for making these judgements will be discussed in detail in the following sections.

Domain of Applicability

Life Stage Applicability

Life Stage	Evidence
Embryo	High
Juvenile	High
Adult	High

Taxonomic Applicability

Term	Scientific Term	Evidence	Links
zebrafish	Danio rerio	High	NCBI
Lemna minor	Lemna minor	Moderate	NCBI
human	Homo sapiens	Moderate	NCBI
mouse	Mus musculus	High	NCBI
rat	Rattus norvegicus	High	NCBI
Caenorhabditis elegans	Caenorhabditis elegans	Moderate	NCBI

Sex Applicability

Sex	Evidence
Unspecific	High

The taxonomic application domain of the AOP potential covers all animals, plants and some microorganisms such as fungus and protists, as mitochondrial OXPHOS is highly conserved in eukaryotes (Roger 2017).

The life stage applicability domain of the AOP mainly contains embryos and juveniles, as growth is more relevant to developing organisms. It should be noted that fully grown adults are also susceptible to uncouplers, as tissue/organ (e.g., adipose tissue) growth and regeneration still occur in adults (Yun 2015; Demine 2019). Classical uncouplers such as 2,4-DNP have been reported to cause weight loss in adult humans (Grundlingh 2011). In fact, 2,4-DNP was sold for weight loss until its legal sale was banned over toxicity and abuse concerns (Baker 2020). These suggest that adults are in the applicability domain of this AOP.

The sex applicability domain of the AOP is unspecific, as the AOP is mainly targeting growth effects in sexually immature organisms and the KEs are therefore harmonized between male and females. However, male and females may have different sensitivities to OXPHOS uncoupling, as strategies for allocating energy for developmental processes may be gender specific (Demarest 2015).

The chemical applicability domain of the AOP mainly includes weak acids, such as phenols, benzimidazoles, N-phenylanthranilates, salicylanilides, phenylhydrazones, salicylic acids, acyldithiocarbazates, cumarines, and aromatic amines, which are well-known protonophoric uncouplers. Uncouplers typically have properties as both weak acids and hydrophobic substances. As weak acids, they are capable of gaining and losing an electron. As hydrophobic substances, they are capable of distributing a negative charge over a number of atoms (often by π-orbitals which delocalize a proton's charge when it attaches to the molecule),

so that they can diffuse back and forth across the inner mitochondrial membrane in either the charged or uncharged state, thus moving protons back across the concentration gradient generated by the electron transport chain. Classical uncouplers, such as 2,4-dinitrophenol (2,4-DNP), carbonyl cyanide-p-trifluoromethoxyphenyl hydrazone (FCCP), carbonyl cyanide m-chlorophenyl hydrazone (CCCP), pentachlorophenol (PCP), 3,5-dichlorophenol (3,5-DCP), 6-sec-butyl-2,4-dinitrophenol (dinoseb), SF 6847 (3,5-di-t-butyl-4-hydroxybenzylidinemalononitrile) have been widely used as positive controls in (eco)toxicological tests, whereas the hazards of "new" uncouplers, such as triclosan, emodin and metabolites of polybrominated diphenyl ethers (PBDEs) are also under extensive assessments. Other types of uncouplers that are SH-reactive chemicals or hydrophobic ions may also be in the applicability domain of this AOP. A number of potential uncouplers have been identified by in silico (Russom 1997; Schultz 1997; Naven 2012; Dreier 2019; Troger 2020) and in vitro (Escher 2002; Attene-Ramos 2013; Attene-Ramos 2015; Xia 2018) approaches, and are considered in the chemical applicability domain of the AOP.

Essentiality of the Key Events

**Support for Essentiality of KEs**	**Defining Question**	**What is the impact on downstream KEs and/or the AO if an upstream KE is modified or prevented?**
	**High**	**Direct evidence from specifically designed experimental studies illustrating prevention or impact on downstream KEs and/or the AO if upstream KEs are blocked or modified.**
	**Moderate**	**Indirect evidence that modification of one or more upstream KEs is associated with a corresponding (increase or decrease) in the magnitude or frequency of downstream KEs.**
	**Low**	**No or contradictory experimental evidence of the essentiality of any of the KEs.**
**Event 1446:**<br><br>**(Decrease, Coupling of OXPHOS)**	**Essentiality of Event 1446 is** high.<br><br>**Rationale:** There is direct evidence from several specifically designed studies showing that removal of an uncoupler from exposure, or addition of a "recoupler" can lead to recovery of the mitochondrial membrane potential (MMP) and total ATP caused by the uncoupler.<br><br>**Evidence**:<br>• Removal of the classical uncoupler carbonyl cyanide-p-trifluoromethoxyphenylhydrazone (FCCP) led to recovery of both MMP and ATP in rat cerebellar granule cells (Weisová 2012).<br>• In the red abalone (*Haliotis rufescens*) larvae, removal of the uncoupler pentachlorophenol also led to recovery of the ATP level (Shofer 2002).<br>• Addition of the recoupler GDP led to a rapid increase in ATP/ADP ratio in isolated guinea pig brown-adipose-tissue mitochondria where high activities of natural coupling by the UCPs were expected (Rafael 1976).<br>• Addition of octanoate to 2,4-DNP exposed rat hepatocytes mitigated the uncoupling effect and partial restored the ATP/ADP ratio (Sibille 1995).<br>• Removal of FCCP led to recovery from FCCP-mediated MMP and ATP reduction in Swiss mouse embryos (Zander-Fox 2015).	
**Event 1771:**<br><br>**(Decrease, ATP pool)**	**Essentiality of Event 1771 is** moderate.<br><br>**Rationale:** There is limited direct evidence from specifically designed studies. However, multiple lines of indirect evidence show that modulation of ATP levels by uncouplers can also lead to corresponding changes in cell proliferation.<br><br>**Evidence**:<br>• Addition of emodin blunted ATP-induced cell proliferation in a concentration-dependent manner in human lung adenocarconoma (A549) cells (Wang 2017), hence providing direct evidence to support the essentiality of this KE.<br>• Positive relationships between uncoupler-mediated ATP depletion and reduced cell proliferation have been documented by multiple studies(Sweet 1999; Fine 2009; Guimarães 2012; Sugiyama 2019).	
**Event 1821:**<br><br>**(Decrease, Cell proliferation)**	**Essentiality of Event 1821 is** moderate.<br><br>**Rationale:** There is no direct evidence from specifically designed studies to support this KE. However, there are multiple lines of indirect evidence showing positive relationships between cell proliferation and growth.<br><br>**Evidence**:<br>• Indirect evidence can be obtained from a limited number of relevant studies showing a positive role of cell proliferation in mammalian tumor (Figarola 2018) zebrafish embryo growth(Bestman 2015).	

**Inconsistencies & uncertainties**	There is an uncertainty related to KE1446 that mild uncoupling of OXPHOS may also increase the ATP pool in some cases (Desquiret 2006), possibly as a compensatory response. The underlying mechanism remains to be further elucidated.

Weight of Evidence Summary

Biological plausibility

**Support for Biological Plausibility of KERs**	**Defining Question**	**Is there a mechanistic (i.e., structural or functional) relationship between KE_{up} and KE_{down} consistent with established biological knowledge?**
	**High**	**Extensive understanding based on extensive previous documentation and broad acceptance -Established mechanistic basis.**
	**Moderate**	**The KER is plausible based on analogy to accepted biological relationships but scientific understanding is not completely established.**
	**Low**	**There is empirical support for a statistical association between KEs, but the structural or functional relationship between them is not understood.**
**Relationship 2203:**<br><br>**(Decrease, Coupling of OXPHOS leads to Decrease, ATP pool)**	**Biological Plausibility of Relationship 2203 is** high.<br><br>**Rationale:** In eukaryotic cells, the major metabolic pathways responsible for ATP production are OXPHOS, citric acid (TCA) cycle, glycolysis and photosynthesis. Oxidative phosphorylation is much (theoretically 15-18 times) more efficient than the rest due to high energy derived from oxygen during aerobic respiration (Schmidt-Rohr 2020). As the ATP level is relatively balanced between production and consumption (Bonora 2012), ATP depletion is a plausible consequence of reduced ATP synthetic efficiency following uncoupling of OXPHOS.	
**Relationship 2204:**<br><br>**(Decrease, ATP pool leads to Decrease, Cell proliferation)**	**Biological Plausibility of Relationship 2204 is** high.<br><br>**Rationale:** Cell proliferation is a well-known ATP-dependent process. Cell division processes, such as the mitotic cell cycle uses ATP for chromosome movements and DNA replication (Kingston 1999). The synthetic processes of major cellular components that are necessary for cell structure and growth, such as proteins and lipids, also require sufficient ATP supply (Bonora 2012). Depletion of ATP therefore has a negative impact on these processes.	
**Relationship 2205:**<br><br>**(Decrease, Cell proliferation leads to Decrease, Growth)**	**Biological Plausibility of Relationship 2205 is** high.<br><br>**Rationale:** The biological causality between cell proliferation and growth has also been well established. It is commonly accepted that the size of an organism, organ or tissue is dependent on the total number and volume of the cells it contains, and the amount of extracellular matrix and fluids (Conlon 1999). Impairment to cell proliferation can logically affect tissue and organismal growth.	
**Inconsistencies & uncertainties**	There are currently no inconsistencies and uncertainties identified by the authors.	

Empirical support

**Empirical Support for KERs**	**Defining Question**	**Does KE_{up} occur at lower doses and earlier time points than KE down and at the same dose of stressor, is the incidence of KE_{up} >than that for KE_{down}? Are there inconsistencies in empirical support across taxa, species and stressors that don't align with expected pattern for hypothesized AOP?**
	**High**	**Multiple studies showing dependent change in both events following exposure to a wide range of specific stressors. (Extensive evidence for temporal, dose- response and incidence concordance) and no or few critical data gaps or conflicting data.**
	**Moderate**	**Demonstrated dependent change in both events following exposure to a small number of specific stressors and some evidence inconsistent with expected pattern that can be explained by factors such as experimental design, technical considerations, differences among laboratories, etc.**
	**Low**	**Limited or no studies reporting dependent change in both events following exposure to a specific stressor (i.e., endpoints never measured in the same study or not at all); and/or significant inconsistencies in empirical support across taxa and species that don't align with expected pattern for hypothesised AOP.**
**Relationship 2203:**<br><br>**(Decrease, Coupling of OXPHOS leads to Decrease, ATP pool)**	**Empirical support of Relationship 2203 is** high.<br><br>**Rationale:** The majority of relevant studies show good incidence, temporal and/or dose concordance in different organisms and cell types after exposure to known uncouplers, with relatively few exceptions (see the Relationship 2203 page and concordance table for detailed evidence).	
**Relationship 2204:**	**Empirical support of Relationship 2204 is** moderate.	

Relationship 2204:<br><br>**(Decrease, ATP pool leads to Decrease, Cell proliferation)**	**Empirical support of Relationship 2204 is** moderate.<br><br>**Rationale:** Although only a few studies were found to be relevant, incidence concordance was found for mammalian cells (see the Relationship 2204 page and concordance table for detailed evidence).
**Relationship 2205:**<br><br>**(Decrease, Cell proliferation leads to Decrease, Growth)**	**Empirical support of Relationship 2205 is** low.<br><br>**Rationale:** This KER was included in a very limited number of studies, as it addresses effects occurring at the apical level that *in vitro* studies cannot cover. There is one zebrafish study reporting concordant relationship between reduced cell proliferation and embryo growth with some inconsistencies (see the Relationship 2205 page and concordance table for detailed evidence).
**Inconsistencies & uncertainties**	There are some inconsistencies regarding temporal and dose concordance:<br><br>• A significant decrease followed by a significant increase of total ATP (KE1) was observed in human RD cells during a 48h exposure to the uncoupler FCCP(Kuruvilla 2003), possibly due to the enhancement of other ATP synthetic pathways (e.g., glycolysis) as a compensatory action to impaired OXPHOS (Jose 2011).<br>• In zebrafish embryos exposed to 2,4-DNP, significant growth inhibition (AO) was identified after 21h, whereas non-significant reductions in ATP (KE1) and cell proliferation (KE2) were reported(Bestman 2015).

Quantitative Consideration

<table>
<tr><td rowspan="3">Quantitative understanding of the KERs</td><td>High</td><td>Change in $KE_{downstream}$ can be precisely predicted based on a relevant measure of $KE_{upstream}$. Uncertainty in the quantitative prediction can be precisely estimated from the variability in the relevant measure of $KE_{upstream}$. Known modulating factors and feedback/feedforward mechanisms are accounted for in the quantitative description. There is evidence that the quantitative relationship between the KEs generalises across the relevant applicability domain of the KER.</td></tr>
<tr><td>Moderate</td><td>Change in $KE_{downstream}$ can be precisely predicted based on a relevant measure of $KE_{upstream}$. Uncertainty in the quantitative prediction is influenced by factors other than the variability in the relevant measure of $KE_{upstream}$. Quantitative description does not account for all known modulating factors and/or known feedback/feedforward mechanisms. The quantitative relationship has only been demonstrated for a subset of the overall applicability domain of the KER (e.g., based on a single species).</td></tr>
<tr><td>Low</td><td>Only a qualitative or semi-quantitative prediction of the change in $KE_{downstream}$ can be determined from a measure of $KE_{upstream}$. Known modulating factors and/or known feedback/feedforward mechanisms are not accounted for. The quantitative relationship has only been demonstrated for a narrow subset of the overall applicability domain of the KER (e.g., based on a single species).</td></tr>
<tr><td>Relationship 2203:

(Decrease, Coupling of OXPHOS leads to Decrease, ATP pool)</td><td colspan="2">Quantitative understanding of Relationship 2203 is high.

Rationale: The theoretical quantitative relationship between OXPHOS and ATP yield has been well established. There are also published computational/mathematical models in which modulating factors known to affect OXPHOS and ATP synthesis are considered.

Evidence:

• A biophysical computational model developed for mitochondrial respiration and OXPHOS (Beard 2005).
• Continuous development of the mitochondrial energy transduction models since 1967 (Schmitz 2011).
• A comprehensive mathematical model developed for OXPHOS and ATP production under different physiological and pathological conditions (Heiske 2017).
• A comprehensive analysis of the quantitative relationships between protonmotive force, ATP synthase rotation, ATP synthesis and hydrolysis (Kubo 2020).
• A regression based response-response relationship for uncoupling of OXPHOS and ATP depletion (Song 2020).</td></tr>
<tr><td>Relationship 2204:

(Decrease, ATP pool leads to Decrease,</td><td colspan="2">Quantitative understanding of Relationship 2204 is moderate.

Rationale: The total ATP level has been used as an indicator of cell proliferation. Several studies have reported the quantitative relationships between the two events, as well as a threshold value for KE1 to trigger KE2. However, not all modulating factors have been accounted and no well-established computational/mathematical models are found.

Evidence:</td></tr>
</table>

**Cell proliferation)**	• Quantitative understanding of ATP level, cell viability and colony growth (Ahmann 1987).<br>• Quantitative relationship between ATP level and cell proliferation (Crouch 1993).<br>• Thresholds for ATP depletion (85-90% reduction) to determine cell cycle arrest (<85-90%) or cell death (>85-90%) (Nieminen 1994).
**Relationship 2205:**<br><br>**(Decrease, Cell proliferation leads to Decrease, Growth)**	**Quantitative understanding of Relationship 2205 is** moderate.<br><br>**Rationale:** Multiple mathematical models describing the quantitative relationships between cell proliferation and tissue growth exist for both animals (Binder 2008) and plants (Mosca 2018). There are also numerous models that are specifically developed for predicting tumor growth based on the proliferation rate (Jarrett 2018). However, there is currently a lack of quantitative model to link cell proliferation and individual growth in the presence of uncouplers.<br><br>Evidence:<br><br>• A mathematical model developed for describing the quantitative relationship between cell proliferation and tissue growth (Binder 2008).<br>• A mathematical model developed for cell division and plant tissue growth (Mosca 2018).<br>• Multiple mathematical models developed for cell proliferation and tumor growth (Jarrett 2018).

Considerations for Potential Applications of the AOP (optional)

The present AOP has several potential applications. First, the AOP anchors a recognized endpoint of regulatory concern (i.e., growth), at least in OECD member countries, and is directly relevant for a number of OECD test guidelines (e.g., TG206, 208, 201, 210, 211, 212, ,215, 221, 228 and 241). These guidelines cover a diversity of taxonomic groups including birds, fish, amphibians, terrestrial plants, aquatic plants and algae, and various invertebrates. Second, the AOP anchors an important molecular initiating event (e.g., uncoupling of oxidative phosphorylation) and can be used to support several initiatives (e.g., Tox21 and ToxCast) for identification of mitochondrial toxicants. The present AOP helps establish the utility of such assays for identifying chemicals with potential to cause growth impacts. Third, three out of four key events in this AOP can be measured using high-throughput *in vitro* assays, hence offering a tiered testing strategy (i.e., *in silico→in vitro→in vivo*) or integrated approaches to testing and assessment (IATA) for efficient screening, classification and assessment of potential mitochondrial uncouplers and growth-regulating chemicals. Finally, the quantitative relationships of the key events in this AOP have been relatively well defined, allowing it to be further developed into quantitative prediction models for higher tier assessments. This is a range of potential applications that were conceived during the development of the present AOP. However, it is neither an exhaustive list of potential applications, nor can explicit examples of these applications in practice be cited at this time.

We invite users of this AOP to share their applications of this AOP via the Discussion so that practical examples of use can be added.

References

Ahmann FR, Garewal HS, Schifman R, Celniker A, Rodney S. 1987. Intracellular adenosine triphosphate as a measure of human tumor cell viability and drug modulated growth. In Vitro Cellular & Developmental Biology 23:474-480. DOI: 10.1007/BF02628417.

Attene-Ramos MS, Huang R, Sakamuru S, Witt KL, Beeson GC, Shou L, Schnellmann RG, Beeson CC, Tice RR, Austin CP, Xia M. 2013. Systematic study of mitochondrial toxicity of environmental chemicals using quantitative high throughput screening. Chemical Research in Toxicology 26:1323-1332. DOI: 10.1021/tx4001754.

Attene-Ramos MS, Huang RL, Michael S, Witt KL, Richard A, Tice RR, Simeonov A, Austin CP, Xia MH. 2015. Profiling of the Tox21 chemical collection for mitochondrial function to identify compounds that acutely decrease mitochondrial membrane potential. Environ Health Persp 123:49-56. DOI: 10.1289/ehp.1408642.

Baker J, Baker M. 2020. Case Report: A Hyperthermic Death from the Diet Pill DNP. ACEP Now. Available from: https://www.acepnow.com/article/case-report-a-hyperthermic-death-from-the-diet-pill-dnp/

Beard DA. 2005. A biophysical model of the mitochondrial respiratory system and oxidative phosphorylation. PLOS Computational Biology 1:e36. DOI: 10.1371/journal.pcbi.0010036.

Becker RA, Ankley GT, Edwards SW, Kennedy SW, Linkov I, Meek B, Sachana M, Segner H, Van der Burg B, Villeneuve DL, Watanabe H, Barton-Maclaren TS. 2015. Increasing scientific confidence in Adverse Outcome Pathways: application of tailored Bradford-Hill considerations for evaluating weight of evidence. Regul Toxicol Pharm 72:514-537. DOI: 10.1016/j.yrtph.2015.04.004.

Bestman JE, Stackley KD, Rahn JJ, Williamson TJ, Chan SS. 2015. The cellular and molecular progression of mitochondrial dysfunction induced by 2,4-dinitrophenol in developing zebrafish embryos. Differentiation 89:51-69. DOI: 10.1016/j.diff.2015.01.001.

Binder BJ, Landman KA, Simpson MJ, Mariani M, Newgreen DF. 2008. Modeling proliferative tissue growth: a general approach and an avian case study. Phys Rev E Stat Nonlin Soft Matter Phys 78:031912. DOI: 10.1103/PhysRevE.78.031912.

Bonora M, Patergnani S, Rimessi A, De Marchi E, Suski JM, Bononi A, Giorgi C, Marchi S, Missiroli S, Poletti F, Wieckowski MR,

Pinton P. 2012. ATP synthesis and storage. Purinergic Signalling 8:343-357. DOI: 10.1007/s11302-012-9305-8.

Conlon I, Raff M. 1999. Size control in animal development. Cell 96:235-244. DOI: 10.1016/s0092-8674(00)80563-2.

Crouch SPM, Kozlowski R, Slater KJ, Fletcher J. 1993. The use of ATP bioluminescence as a measure of cell proliferation and cytotoxicity. Journal of Immunological Methods 160:81-88. DOI: https://doi.org/10.1016/0022-1759(93)90011-U.

Demarest TG, McCarthy MM. 2015. Sex differences in mitochondrial (dys)function: Implications for neuroprotection. Journal of Bioenergetics and Biomembranes 47:173-188. DOI: 10.1007/s10863-014-9583-7.

Demine S, Renard P, Arnould T. 2019. Mitochondrial uncoupling: a key controller of biological processes in physiology and diseases. Cells 8. DOI: 10.3390/cells8080795.

Desquiret V, Loiseau D, Jacques C, Douay O, Malthièry Y, Ritz P, Roussel D. 2006. Dinitrophenol-induced mitochondrial uncoupling in vivo triggers respiratory adaptation in HepG2 cells. Biochimica et Biophysica Acta (BBA) - Bioenergetics 1757:21-30. DOI: https://doi.org/10.1016/j.bbabio.2005.11.005.

Dreier DA, Denslow ND, Martyniuk CJ. 2019. Computational in vitro toxicology uncovers chemical structures impairing mitochondrial membrane potential. J Chem Inf Model 59:702-712. DOI: 10.1021/acs.jcim.8b00433.

Escher BI, Schwarzenbach RP. 2002. Mechanistic studies on baseline toxicity and uncoupling of organic compounds as a basis for modeling effective membrane concentrations in aquatic organisms. Aquatic Sciences 64:20-35. DOI: 10.1007/s00027-002-8052-2.

Figarola JL, Singhal J, Singhal S, Kusari J, Riggs A. 2018. Bioenergetic modulation with the mitochondria uncouplers SR4 and niclosamide prevents proliferation and growth of treatment-naive and vemurafenib-resistant melanomas. Oncotarget 9:36945-36965. DOI: 10.18632/oncotarget.26421.

Fine EJ, Miller A, Quadros EV, Sequeira JM, Feinman RD. 2009. Acetoacetate reduces growth and ATP concentration in cancer cell lines which over-express uncoupling protein 2. Cancer Cell International 9:14. DOI: 10.1186/1475-2867-9-14.

Grundlingh J, Dargan PI, El-Zanfaly M, Wood DM. 2011. 2,4-dinitrophenol (DNP): a weight loss agent with significant acute toxicity and risk of death. J Med Toxicol 7:205-212. DOI: 10.1007/s13181-011-0162-6.

Guimarães EL, Best J, Dollé L, Najimi M, Sokal E, van Grunsven LA. 2012. Mitochondrial uncouplers inhibit hepatic stellate cell activation. BMC Gastroenterology 12:68. DOI: 10.1186/1471-230X-12-68.

Heiske M, Letellier T, Klipp E. 2017. Comprehensive mathematical model of oxidative phosphorylation valid for physiological and pathological conditions. The FEBS Journal 284:2802-2828. DOI: https://doi.org/10.1111/febs.14151.

Jarrett AM, Lima EABF, Hormuth DA, McKenna MT, Feng X, Ekrut DA, Resende ACM, Brock A, Yankeelov TE. 2018. Mathematical models of tumor cell proliferation: A review of the literature. Expert Review of Anticancer Therapy 18:1271-1286. DOI: 10.1080/14737140.2018.1527689.

Jose C, Bellance N, Rossignol R. 2011. Choosing between glycolysis and oxidative phosphorylation: A tumor's dilemma? Biochimica et Biophysica Acta (BBA) - Bioenergetics 1807:552-561. DOI: https://doi.org/10.1016/j.bbabio.2010.10.012.

Kingston RE, Narlikar GJ. 1999. ATP-dependent remodeling and acetylation as regulators of chromatin fluidity. Genes Dev 13:2339-2352. DOI: 10.1101/gad.13.18.2339.

Kubo S, Niina T, Takada S. 2020. Molecular dynamics simulation of proton-transfer coupled rotations in ATP synthase FO motor. Scientific Reports 10:8225. DOI: 10.1038/s41598-020-65004-1.

Kuruvilla S, Qualls CW, Jr., Tyler RD, Witherspoon SM, Benavides GR, Yoon LW, Dold K, Brown RH, Sangiah S, Morgan KT. 2003. Effects of minimally toxic levels of carbonyl cyanide P-(trifluoromethoxy) phenylhydrazone (FCCP), elucidated through differential gene expression with biochemical and morphological correlations. Toxicol Sci 73:348-361. DOI: 10.1093/toxsci/kfg084.

Liberman EA, Topaly VP, Tsofina LM, Jasaitis AA, Skulachev VP. 1969. Mechanism of coupling of oxidative phosphorylation and the membrane potential of mitochondria. Nature 222:1076-1078. DOI: 10.1038/2221076a0.

Mosca G, Adibi, M., Strauss, S., Runions, A., Sapala, A., Smith, R.S. 2018. Modeling Plant Tissue Growth and Cell Division. In Morris R., ed, Mathematical Modelling in Plant Biology. Springer, Cham.

Naven RT, Swiss R, Klug-Mcleod J, Will Y, Greene N. 2012. The development of structure-activity relationships for mitochondrial dysfunction: Uncoupling of oxidative phosphorylation. Toxicol Sci 131:271-278. DOI: 10.1093/toxsci/kfs279.

Nieminen AL, Saylor AK, Herman B, Lemasters JJ. 1994. ATP depletion rather than mitochondrial depolarization mediates hepatocyte killing after metabolic inhibition. Am J Physiol 267:C67-74. DOI: 10.1152/ajpcell.1994.267.1.C67.

OECD. 2018. Users' Handbook supplement to the Guidance Document for developing and assessing Adverse Outcome Pathways. -OECD Series on Adverse Outcome Pathways, No. 1. OECD Publishing, Paris.

Rafael J, Wrabetz E. 1976. Brown adipose tissue mitochondria recoupling caused by substrate level phosphorylation and extramitochondrial adenosine phosphates. European Journal of Biochemistry 61:551-561. DOI: https://doi.org/10.1111/j.1432-

1033.1976.tb10050.x.

Ramaiah A, Hathaway JA, Atkinson DE. 1964. Adenylate as a metabolic regulator. Effect on yeast phosphofructokinase kinetics. J Biol Chem 239:3619-3622.

Roger AJ, Munoz-Gomez SA, Kamikawa R. 2017. The origin and diversification of mitochondria. Curr Biol 27:R1177-R1192. DOI: 10.1016/j.cub.2017.09.015.

Russom CL, Bradbury SP, Broderius SJ, Hammermeister DE, Drummond RA. 1997. Predicting modes of toxic action from chemical structure: Acute toxicity in the fathead minnow (Pimephales promelas). Environ Toxicol Chem 16:948-967. DOI: https://doi.org/10.1002/etc.5620160514.

Schmidt-Rohr K. 2020. Oxygen is the high-energy molecule powering complex multicellular life: fundamental corrections to traditional bioenergetics. ACS Omega 5:2221-2233. DOI: 10.1021/acsomega.9b03352.

Schmitz JPJ, Vanlier J, van Riel NAW, Jeneson JAL. 2011. Computational modeling of mitochondrial energy transduction. 39:363-377. DOI: 10.1615/CritRevBiomedEng.v39.i5.20.

Schultz TW, Cronin MTD. 1997. Quantitative structure-activity relationships for weak acid respiratory uncouplers to Vibrio fisheri. Environ Toxicol Chem 16:357-360. DOI: https://doi.org/10.1002/etc.5620160235.

Shofer SL, Tjeerdema RS. 2002. Sublethal effects of pentachlorophenol in abalone (Haliotis rufescens) veliger larvae as measured by (31)P-NMR. Ecotoxicology and Environmental Safety 51:155-160. DOI: https://doi.org/10.1006/eesa.2002.2141.

Sibille B, Keriel C, Fontaine E, Catelloni F, Rigoulet M, Leverve XM. 1995. Octanoate affects 2,4-dinitrophenol uncoupling in intact isolated rat hepatocytes. European Journal of Biochemistry 231:498-502. DOI: 10.1111/j.1432-1033.1995.tb20724.x.

Song Y, Xie L, Lee Y, Tollefsen KE. 2020. De novo development of a quantitative adverse outcome pathway (qAOP) network for ultraviolet B (UVB) radiation using targeted laboratory tests and automated data mining. Environmental Science & Technology 54:13147-13156. DOI: 10.1021/acs.est.0c03794.

Sugiyama Y, Shudo T, Hosokawa S, Watanabe A, Nakano M, Kakizuka A. 2019. Emodin, as a mitochondrial uncoupler, induces strong decreases in adenosine triphosphate (ATP) levels and proliferation of B16F10 cells, owing to their poor glycolytic reserve. Genes to Cells 24:569-584. DOI: https://doi.org/10.1111/gtc.12712.

Sweet S, Singh G. 1999. Changes in mitochondrial mass, membrane potential, and cellular adenosine triphosphate content during the cell cycle of human leukemic (HL-60) cells. Journal of Cellular Physiology 180:91-96. DOI: https://doi.org/10.1002/(SICI)1097-4652(199907)180:1<91::AID-JCP10>3.0.CO;2-6.

Terada H. 1990. Uncouplers of oxidative phosphorylation. Environ Health Perspect 87:213-218. DOI: 10.1289/ehp.9087213.

Troger F, Delp J, Funke M, van der Stel W, Colas C, Leist M, van de Water B, Ecker GF. 2020. Identification of mitochondrial toxicants by combined in silico and in vitro studies – A structure-based view on the adverse outcome pathway. Computational Toxicology 14:100123. DOI: https://doi.org/10.1016/j.comtox.2020.100123.

Wang X, Li L, Guan R, Zhu D, Song N, Shen L. 2017. Emodin inhibits ATP-induced proliferation and migration by suppressing P2Y receptors in human lung adenocarcinoma cells. Cellular Physiology and Biochemistry 44:1337-1351. DOI: 10.1159/000485495.

Weisová P, Anilkumar U, Ryan C, Concannon CG, Prehn JHM, Ward MW. 2012. 'Mild mitochondrial uncoupling' induced protection against neuronal excitotoxicity requires AMPK activity. Biochimica et Biophysica Acta (BBA) - Bioenergetics 1817:744-753. DOI: https://doi.org/10.1016/j.bbabio.2012.01.016.

Xia M, Huang R, Shi Q, Boyd WA, Zhao J, Sun N, Rice JR, Dunlap PE, Hackstadt AJ, Bridge MF, Smith MV, Dai S, Zheng W, Chu PH, Gerhold D, Witt KL, DeVito M, Freedman JH, Austin CP, Houck KA, Thomas RS, Paules RS, Tice RR, Simeonov A. 2018. Comprehensive analyses and prioritization of Tox21 10K chemicals affecting mitochondrial function by in-depth mechanistic studies. Environ Health Perspect 126:077010. DOI: 10.1289/EHP2589.

Yun MH. 2015. Changes in regenerative capacity through lifespan. International Journal of Molecular Sciences 16:25392-25432.

Zander-Fox DL, Fullston T, McPherson NO, Sandeman L, Kang WX, Good SB, Spillane M, Lane M. 2015. Reduction of mitochondrial function by FCCP during mouse cleavage stage embryo culture reduces birth weight and impairs the netabolic health of offspring1. Biology of Reproduction 92. DOI: 10.1095/biolreprod.114.123489.

Appendix 1

List of MIEs in this AOP

Event: 1446: Decrease, Coupling of oxidative phosphorylation

Short Name: Decrease, Coupling of OXPHOS

Key Event Component

Process	Object	Action
proton binding	mitochondrion	increased
oxidative phosphorylation uncoupler activity	mitochondrion	increased
regulation of mitochondrial membrane potential	mitochondrion	decreased

AOPs Including This Key Event

AOP ID and Name	Event Type
Aop:267 - Uncoupling of oxidative phosphorylation leading to growth inhibition via increased lipid peroxidation	MolecularInitiatingEvent
Aop:263 - Uncoupling of oxidative phosphorylation leading to growth inhibition via decreased cell proliferation	MolecularInitiatingEvent
Aop:264 - Uncoupling of oxidative phosphorylation leading to growth inhibition via increased cell death	MolecularInitiatingEvent
Aop:265 - Uncoupling of oxidative phosphorylation leading to growth inhibition via decreased lipid storage	MolecularInitiatingEvent
Aop:266 - Uncoupling of oxidative phosphorylation leading to growth inhibition via oxidative DNA damage	MolecularInitiatingEvent
Aop:268 - Uncoupling of oxidative phosphorylation leading to growth inhibition via increased protein oxidation	MolecularInitiatingEvent

Stressors

Name
2,4-Dinitrophenol
Carbonyl cyanide-p-trifluoromethoxyphenylhydrazone
Carbonyl cyanide m-chlorophenyl hydrazone
Pentachlorophenol
Triclosan
Emodin
Malonoben

Biological Context

Level of Biological Organization
Molecular

Cell term

Cell term
cell

Evidence for Perturbation by Stressor

Overview for Molecular Initiating Event

Decreased coupling of oxidative phosphorylation can be directly triggered by “uncouplers” as a molecular initiating event.

- Most of the chemical uncouplers are protonophores, a type of proton binders that can translocate protons across membranes.

These protonophores share several common structural characteristics, such as bulky hydrophobic moiety, an acid dissociable group and a strong electron-withdrawing group (Terada 1990). Weak acids such as phenols, benzimidazoles and salicylic acids are considered potential protonophores.
- Classical uncouplers, such as carbonyl cyanide-p-trifluoromethoxyphenylhydrazone (FCCP), carbonyl cyanide m-chlorophenyl hydrazone (CCCP), 2,4-dinitrophenol (DNP), pentachlorophenol (PCP) and SF-6847 (Terada 1990).
- Newer uncouplers, such as triclosan (Shim 2016; Weatherly 2016), emodin (Sugiyama 2019), and hydroxylated polybrominated diphenyl ethers (PBDEs) (Legradi 2014) have been widely investigated in vertebrates.
- Computational predictions based on quantitative structure-activity relationships (Russom 1997; Schultz 1997; Naven 2012; Dreier 2019; Troger 2020) and in vitro high-throughput screening (Escher 2002; Attene-Ramos 2013; Attene-Ramos 2015; Xia 2018) have facilitated the identification and classification of potential uncouplers from a large list of chemicals.

Domain of Applicability

Taxonomic Applicability

Term	Scientific Term	Evidence	Links
zebrafish	Danio rerio	High	NCBI
human	Homo sapiens	High	NCBI
mouse	Mus musculus	High	NCBI
rat	Rattus norvegicus	High	NCBI
Lemna minor	Lemna minor	High	NCBI

Life Stage Applicability

Life Stage	Evidence
Embryo	High
Juvenile	High
Adult, reproductively mature	Moderate

Sex Applicability

Sex	Evidence
Unspecific	High

Taxonomic applicability domain

This key event is in general considered applicable to most eukaryotes, as the mitochondrion and oxidative phosphorylation are highly conserved (Roger 2017).

Life stage applicability domain

This key event is considered applicable to all life stages, as ATP synthesis by oxidative phosphorylation is an essential biological process for most living organisms.

Sex applicability domain

This key event is considered sex-unspecific, as both males and females use oxidative phosphorylation as a main process to generate ATP.

Key Event Description

Decreased coupling of oxidative phosphorylation (OXPHOS), or uncoupling of OXPHOS, describes dissipation of protonmotive force (PMF) across the inner mitochondrial membrane (IMM) by environmental stressors. In eukaryotes, the mitochondrial electron transport chain mediates a series of redox reactions to create a PMF across the IMM. The PMF is used as energy to drive adenosine triphosphate (ATP) synthesis through phosphorylation of adenosine diphosphate (ADP). These processes are coupled and referred to as OXPHOS. A number of chemicals can dissipate the PMF, leading to uncoupling of OXPHOS. This key event describes the main outcome of the interactions between an uncoupler and the transmembrane PMF. An uncoupler can bind to a proton in the mitochondrial inter membrane space, transport the proton to the matrix side of the IMM, release the proton and move back to the inter membrane space. These processes are repeated until the transmembrane PMF is dissipated. This KE is therefore a lumped term of these processes and represents the final consequence of the interactions.

How it is Measured or Detected

Uncoupling of oxidative phosphorylation can be indicated by reduced mitochondrial membrane potential, increased proton leak and/or increased oxygen consumption rate.

- Mitochondrial membrane potential can be determined using ToxCast high-throughput screening bioassays such as “APR_HepG2_MitoMembPot”, “APR_Hepat_MitoFxnI”, and “APR_Mitochondrial_membrane_potential”, and the Tox21 high-throughput screening assay “tox21-mitotox-p1”.
- Mitochondrial membrane potential can also be measured using commercially available fluorescent probes such as TMRM (tetramethylrhodamine, methyl ester, perchlorate), TMRE (tetramethylrhodamine, ethyl ester, perchlorate) and JC-1 (Perry 2011).
- Proton leak and oxygen consumption rate can be measured using a high-resolution respirometry (Affourtit 2018) or a Seahorse XF analyzer (Divakaruni 2014).

References

Affourtit C, Wong H-S, Brand MD. 2018. Measurement of proton leak in isolated mitochondria. In Palmeira CM, Moreno AJ, eds, *Mitochondrial Bioenergetics: Methods and Protocols*. Springer New York, New York, NY, pp 157-170.

Attene-Ramos MS, Huang R, Sakamuru S, Witt KL, Beeson GC, Shou L, Schnellmann RG, Beeson CC, Tice RR, Austin CP, Xia M. 2013. Systematic study of mitochondrial toxicity of environmental chemicals using quantitative high throughput screening. *Chemical Research in Toxicology* 26:1323-1332. DOI: 10.1021/tx4001754.

Attene-Ramos MS, Huang RL, Michael S, Witt KL, Richard A, Tice RR, Simeonov A, Austin CP, Xia MH. 2015. Profiling of the Tox21 chemical collection for mitochondrial function to identify compounds that acutely decrease mitochondrial membrane potential. *Environ Health Persp* 123:49-56. DOI: 10.1289/ehp.1408642.

Divakaruni AS, Paradyse A, Ferrick DA, Murphy AN, Jastroch M. 2014. Chapter Sixteen - Analysis and Interpretation of Microplate-Based Oxygen Consumption and pH Data. In Murphy AN, Chan DC, eds, *Methods in Enzymology*. Vol 547. Academic Press, pp 309-354.

Dreier DA, Denslow ND, Martyniuk CJ. 2019. Computational *in vitro* toxicology uncovers chemical structures impairing mitochondrial membrane potential. *J Chem Inf Model* 59:702-712. DOI: 10.1021/acs.jcim.8b00433.

Escher BI, Schwarzenbach RP. 2002. Mechanistic studies on baseline toxicity and uncoupling of organic compounds as a basis for modeling effective membrane concentrations in aquatic organisms. *Aquatic Sciences* 64:20-35. DOI: 10.1007/s00027-002-8052-2.

Legradi J, Dahlberg A-K, Cenijn P, Marsh G, Asplund L, Bergman Å, Legler J. 2014. Disruption of Oxidative Phosphorylation (OXPHOS) by Hydroxylated Polybrominated Diphenyl Ethers (OH-PBDEs) Present in the Marine Environment. *Environmental Science & Technology* 48:14703-14711. DOI: 10.1021/es5039744.

Naven RT, Swiss R, Klug-Mcleod J, Will Y, Greene N. 2012. The development of structure-activity relationships for mitochondrial dysfunction: Uncoupling of oxidative phosphorylation. *Toxicol Sci* 131:271-278. DOI: 10.1093/toxsci/kfs279.

Perry SW, Norman JP, Barbieri J, Brown EB, Gelbard HA. 2011. Mitochondrial membrane potential probes and the proton gradient: a practical usage guide. *BioTechniques* 50:98-115. DOI: 10.2144/000113610.

Roger AJ, Munoz-Gomez SA, Kamikawa R. 2017. The origin and diversification of mitochondria. *Curr Biol* 27:R1177-R1192. DOI: 10.1016/j.cub.2017.09.015.

Russom CL, Bradbury SP, Broderius SJ, Hammermeister DE, Drummond RA. 1997. Predicting modes of toxic action from chemical structure: Acute toxicity in the fathead minnow (Pimephales promelas). *Environ Toxicol Chem* 16:948-967. DOI: https://doi.org/10.1002/etc.5620160514.

Schultz TW, Cronin MTD. 1997. Quantitative structure-activity relationships for weak acid respiratory uncouplers to Vibrio fisheri. *Environ Toxicol Chem* 16:357-360. DOI: https://doi.org/10.1002/etc.5620160235.

Shim J, Weatherly LM, Luc RH, Dorman MT, Neilson A, Ng R, Kim CH, Millard PJ, Gosse JA. 2016. Triclosan is a mitochondrial uncoupler in live zebrafish. *J Appl Toxicol* 36:1662-1667. DOI: 10.1002/jat.3311.

Sugiyama Y, Shudo T, Hosokawa S, Watanabe A, Nakano M, Kakizuka A. 2019. Emodin, as a mitochondrial uncoupler, induces strong decreases in adenosine triphosphate (ATP) levels and proliferation of B16F10 cells, owing to their poor glycolytic reserve. *Genes to Cells* 24:569-584. DOI: https://doi.org/10.1111/gtc.12712.

Terada H. 1990. Uncouplers of oxidative phosphorylation. *Environ Health Perspect* 87:213-218. DOI: 10.1289/ehp.9087213.

Troger F, Delp J, Funke M, van der Stel W, Colas C, Leist M, van de Water B, Ecker GF. 2020. Identification of mitochondrial toxicants by combined in silico and in vitro studies – A structure-based view on the adverse outcome pathway. *Computational Toxicology* 14:100123. DOI: https://doi.org/10.1016/j.comtox.2020.100123.

Weatherly LM, Shim J, Hashmi HN, Kennedy RH, Hess ST, Gosse JA. 2016. Antimicrobial agent triclosan is a proton ionophore

uncoupler of mitochondria in living rat and human mast cells and in primary human keratinocytes. *Journal of Applied Toxicology* 36:777-789. DOI: https://doi.org/10.1002/jat.3209.

Xia M, Huang R, Shi Q, Boyd WA, Zhao J, Sun N, Rice JR, Dunlap PE, Hackstadt AJ, Bridge MF, Smith MV, Dai S, Zheng W, Chu PH, Gerhold D, Witt KL, DeVito M, Freedman JH, Austin CP, Houck KA, Thomas RS, Paules RS, Tice RR, Simeonov A. 2018. Comprehensive analyses and prioritization of Tox21 10K chemicals affecting mitochondrial function by in-depth mechanistic studies. *Environ Health Perspect* 126:077010. DOI: 10.1289/EHP2589.

List of Key Events in the AOP

Event: 1771: Decrease, Adenosine triphosphate pool

Short Name: Decrease, ATP pool

Key Event Component

Process	Object	Action
ATP biosynthetic process	ATP	decreased

AOPs Including This Key Event

AOP ID and Name	Event Type
Aop:328 - Excessive reactive oxygen species production leading to mortality (2)	KeyEvent
Aop:329 - Excessive reactive oxygen species production leading to mortality (3)	KeyEvent
Aop:264 - Uncoupling of oxidative phosphorylation leading to growth inhibition via increased cell death	KeyEvent
Aop:263 - Uncoupling of oxidative phosphorylation leading to growth inhibition via decreased cell proliferation	KeyEvent
Aop:299 - Excessive reactive oxygen species production leading to population decline via reduced fatty acid beta-oxidation	KeyEvent
Aop:311 - Excessive reactive oxygen species production leading to population decline via mitochondrial dysfunction	KeyEvent
Aop:265 - Uncoupling of oxidative phosphorylation leading to growth inhibition via decreased lipid storage	KeyEvent
Aop:324 - Thermal stress leading to population decline (1)	KeyEvent
Aop:290 - Mitochondrial ATP synthase antagonism leading to growth inhibition (1)	KeyEvent
Aop:291 - Mitochondrial ATP synthase antagonism leading to growth inhibition (2)	KeyEvent
Aop:286 - Mitochondrial complex III antagonism leading to growth inhibition (1)	KeyEvent
Aop:287 - Mitochondrial complex III antagonism leading to growth inhibition (2)	KeyEvent

Stressors

Name
Carbonyl cyanide-p-trifluoromethoxyphenylhydrazone
Carbonyl cyanide m-chlorophenyl hydrazone
2,4-Dinitrophenol
Malonoben
Pentachlorophenol
Triclosan
Emodin

Biological Context

Level of Biological Organization

Cellular

Cell term

Cell term

cell

Domain of Applicability

Taxonomic Applicability

Term	Scientific Term	Evidence	Links
zebrafish	Danio rerio	High	NCBI
human	Homo sapiens	High	NCBI
rat	Rattus norvegicus	High	NCBI
mouse	Mus musculus	High	NCBI

Life Stage Applicability

Life Stage	Evidence
Embryo	High
Juvenile	High
Adult, reproductively mature	Moderate

Sex Applicability

Sex	Evidence
Unspecific	High

Taxonomic applicability domain

This key event is in general considered applicable to all eukaryotes utilizing ATP as a direct source of energy and signaling molecule.

Life stage applicability domain

This key event is considered applicable to all life stages, as all developmental stages require energy supply to maintain necessary physiological processes.

Sex applicability domain

This key event is considered sex-unspecific, as both males and females use ATP as an essential energy molecule.

Key Event Description

Decreased adenosine triphosphate (ATP) pool describes the loss of balance between ATP synthesis and ATP consumption, leading to reduced total ATP. As a primary form of biological energy, ATP is used by many biological processes (Bonora 2012). Decrease in ATP level normally attributes to metabolic disorders in major ATP synthetic pathways, such as mitochondrial oxidative phosphorylation, fatty acid β-oxidation, glycolysis and plant photophosphorylation.

How it is Measured or Detected

-The ATP pool in cells or tissue can be quantified using a well-established ATP bioluminescent assay (Lemasters 1978; Wibom 1990). Assay principles: ATP can react with luciferase and luciferin from firefly and the luminescence emitted from the reaction is proportional to the ATP concentration:

ATP + D-Luciferin + O_2 è Oxyluciferin + AMP + PPi + CO_2 + Light

-ToxCast high-throughput screening bioassays, such as “NCCT_HEK293T_CellTiterGLO” and “NIS_HEK293T_CTG_Cytotoxicity” can be used to measure this KE.

References

Bonora M, Patergnani S, Rimessi A, De Marchi E, Suski JM, Bononi A, Giorgi C, Marchi S, Missiroli S, Poletti F, Wieckowski MR, Pinton P. 2012. ATP synthesis and storage. *Purinergic Signalling* 8:343-357. DOI: 10.1007/s11302-012-9305-8.

Lemasters JJ, Hackenbrock CR. 1978. [4] Firefly luciferase assay for ATP production by mitochondria. *Methods in Enzymology*. Vol 57. Academic Press, pp 36-50.

Wibom R, Lundin A, Hultman E. 1990. A sensitive method for measuring ATP-formation in rat muscle mitochondria. *Scandinavian Journal of Clinical and Laboratory Investigation* 50:143-152. DOI: 10.1080/00365519009089146.

Event: 1821: Decrease, Cell proliferation

Short Name: Decrease, Cell proliferation

Key Event Component

Process	Object	Action
cell proliferation	cell	decreased

AOPs Including This Key Event

AOP ID and Name	Event Type
Aop:263 - Uncoupling of oxidative phosphorylation leading to growth inhibition via decreased cell proliferation	KeyEvent
Aop:290 - Mitochondrial ATP synthase antagonism leading to growth inhibition (1)	KeyEvent
Aop:286 - Mitochondrial complex III antagonism leading to growth inhibition (1)	KeyEvent
Aop:399 - Inhibition of Fyna leading to increased mortality via decreased eye size (Microphthalmos)	KeyEvent

Stressors

Name
2,4-Dinitrophenol
Carbonyl cyanide-p-trifluoromethoxyphenylhydrazone
Carbonyl cyanide m-chlorophenyl hydrazone
Pentachlorophenol
Triclosan
Emodin
Malonoben

Biological Context

Level of Biological Organization
Cellular

Cell term

Cell term

cell
Cell term

Domain of Applicability

Taxonomic Applicability

Term	Scientific Term	Evidence	Links
zebrafish	Danio rerio	High	NCBI
human	Homo sapiens	High	NCBI
rat	Rattus norvegicus	High	NCBI
mouse	Mus musculus	High	NCBI

Life Stage Applicability

Life Stage	Evidence
Embryo	High
Juvenile	High

Sex Applicability

Sex	Evidence
Unspecific	High

Taxonomic applicability domain

This key event is in general applicable to all eukaryotes, as most organisms are known to use cell proliferation to achieve growth.

Life stage applicability domain

This key event is in general applicable to all life stages. As cell proliferation not only occurs in developing organisms, but also in adults.

Sex applicability domain

This key event is sex-unspecific, as both genders use the same cell proliferation mechanisms.

Key Event Description

Decreased cell proliferation describes the outcome of reduced cell division and cell growth. Cell proliferation is considered the main mechanism of tissue and organismal growth (Conlon 1999). Decreased cell proliferation has been associated with abnormal growth-factor signaling and cellular energy depletion (DeBerardinis 2008).

How it is Measured or Detected

Multiple types of *in vitro* bioassays can be used to measure this key event:

- ToxCast high-throughput screening bioassays such as “BSK_3C_Proliferation”, “BSK_CASM3C_Proliferation” and “BSK_SAg_Proliferation” can be used to measure cell proliferation status.
- Commercially available methods such as the well-established 5-bromo-2’-deoxyuridine (BrdU) (Raza 1985; Muir 1990) or 5-ethynyl-2’-deoxyuridine (EdU) assay. Both assays measure DNA synthesis in dividing cells to indicate proliferation status.

References

Conlon I, Raff M. 1999. Size control in animal development. *Cell* 96:235-244. DOI: 10.1016/s0092-8674(00)80563-2.

DeBerardinis RJ, Lum JJ, Hatzivassiliou G, Thompson CB. 2008. The biology of cancer: metabolic reprogramming fuels cell growth and proliferation. *Cell Metabolism* 7:11-20. DOI: https://doi.org/10.1016/j.cmet.2007.10.002.

Muir D, Varon S, Manthorpe M. 1990. An enzyme-linked immunosorbent assay for bromodeoxyuridine incorporation using fixed microcultures. *Analytical Biochemistry* 185:377-382. DOI: https://doi.org/10.1016/0003-2697(90)90310-6.

Raza A, Spiridonidis C, Ucar K, Mayers G, Bankert R, Preisler HD. 1985. Double labeling of S-phase murine cells with bromodeoxyuridine and a second DNA-specific probe. *Cancer Research* 45:2283-2287.

List of Adverse Outcomes in this AOP

Event: 1521: Decrease, Growth

Short Name: Decrease, Growth

Key Event Component

Process	Object	Action
growth	multicellular organism	decreased

AOPs Including This Key Event

AOP ID and Name	Event Type
Aop:263 - Uncoupling of oxidative phosphorylation leading to growth inhibition via decreased cell proliferation	AdverseOutcome
Aop:264 - Uncoupling of oxidative phosphorylation leading to growth inhibition via increased cell death	AdverseOutcome
Aop:265 - Uncoupling of oxidative phosphorylation leading to growth inhibition via decreased lipid storage	AdverseOutcome
Aop:266 - Uncoupling of oxidative phosphorylation leading to growth inhibition via oxidative DNA damage	AdverseOutcome
Aop:267 - Uncoupling of oxidative phosphorylation leading to growth inhibition via increased lipid peroxidation	AdverseOutcome
Aop:268 - Uncoupling of oxidative phosphorylation leading to growth inhibition via increased protein oxidation	AdverseOutcome
Aop:290 - Mitochondrial ATP synthase antagonism leading to growth inhibition (1)	AdverseOutcome
Aop:291 - Mitochondrial ATP synthase antagonism leading to growth inhibition (2)	AdverseOutcome
Aop:286 - Mitochondrial complex III antagonism leading to growth inhibition (1)	AdverseOutcome
Aop:287 - Mitochondrial complex III antagonism leading to growth inhibition (2)	AdverseOutcome
Aop:245 - Reduction in photophosphorylation leading to growth inhibition in aquatic plants	AdverseOutcome

Stressors

Name
2,4-Dinitrophenol
Carbonyl cyanide-p-trifluoromethoxyphenylhydrazone
Carbonyl cyanide m-chlorophenyl hydrazone
Pentachlorophenol
Triclosan
Emodin
Malonoben

Biological Context

Level of Biological Organization
Individual

Domain of Applicability

Taxonomic Applicability

Term	Scientific Term	Evidence	Links
human	Homo sapiens	Moderate	NCBI

Term	Scientific Term	Evidence	Links
rat	Rattus norvegicus	Moderate	NCBI
mouse	Mus musculus	Moderate	NCBI
zebrafish	Danio rerio	High	NCBI
fathead minnow	Pimephales promelas	High	NCBI
Lemna minor	Lemna minor	High	NCBI
Daphnia magna	Daphnia magna	Moderate	NCBI

Life Stage Applicability

Life Stage	Evidence
Embryo	High
Juvenile	High

Sex Applicability

Sex	Evidence
Unspecific	High

Taxonomic applicability domain

This key event is in general applicable to all eukaryotes.

Life stage applicability domain

This key event is applicable to early life stages such as embryo and juvenile.

Sex applicability domain

This key event is sex-unspecific.

Key Event Description

Decreased growth refers to a reduction in size and/or weight of a tissue, organ or individual organism. Growth is normally controlled by growth factors and mainly achieved through cell proliferation (Conlon 1999).

How it is Measured or Detected

Growth can be indicated by measuring weight, length, total volume, and/or total area of a tissue, organ or individual organism.

Regulatory Significance of the AO

Growth is a regulatory relevant chronic toxicity endpoint for almost all organisms. Multiple OECD test guidelines have included growth either as a main endpoint of concern, or as an additional endpoint to be considered in the toxicity assessments. Relevant test guidelines include, but not only limited to:

-Test No. 208: Terrestrial Plant Test: Seedling Emergence and Seedling Growth Test

-Test No. 212: Fish, Short-term Toxicity Test on Embryo and Sac-Fry Stages

-Test No. 215: Fish, Juvenile Growth Test

-Test No. 241: The Larval Amphibian Growth and Development Assay (LAGDA)

-Test No. 201: Freshwater Alga and Cyanobacteria, Growth Inhibition Test

-Test No. 221: Lemna sp. Growth Inhibition Test

-Test No. 228: Determination of Developmental Toxicity to Dipteran Dung Flies (Scathophaga stercoraria L. (Scathophagidae), Musca autumnalis De Geer (Muscidae))

-Test No. 211: Daphnia magna Reproduction Test

References

Conlon I, Raff M. 1999. Size control in animal development. *Cell* 96:235-244. DOI: 10.1016/s0092-8674(00)80563-2.

Appendix 2

List of Key Event Relationships in the AOP

List of Adjacent Key Event Relationships

Relationship: 2203: Decrease, Coupling of OXPHOS leads to Decrease, ATP pool

AOPs Referencing Relationship

AOP Name	Adjacency	Weight of Evidence	Quantitative Understanding
Uncoupling of oxidative phosphorylation leading to growth inhibition via decreased cell proliferation	adjacent	High	High
Uncoupling of oxidative phosphorylation leading to growth inhibition via increased cell death	adjacent		
Uncoupling of oxidative phosphorylation leading to growth inhibition via decreased lipid storage	adjacent		

Evidence Supporting Applicability of this Relationship

Taxonomic Applicability

Term	Scientific Term	Evidence	Links
zebrafish	Danio rerio	High	NCBI
human	Homo sapiens	High	NCBI
rat	Rattus norvegicus	High	NCBI
mouse	Mus musculus	High	NCBI

Life Stage Applicability

Life Stage	Evidence
Embryo	High
Juvenile	High

Sex Applicability

Sex	Evidence
Unspecific	High

Taxonomic applicability

Relationship 2203 is considered applicable to eukaryotes, as mitochondrial oxidative phosphorylation and ATP synthesis are highly conserved in these organisms. Uncoupling of oxidative phosphorylation leading to ATP depletion is a well-documented relationship in many taxa, such as human, rodents and fish.

Sex applicability

Relationship 2203 is considered applicable to all genders, as mitochondrial oxidative phosphorylation and ATP synthesis are fundamental biological processes and are not sex-pecific.

Life-stage applicability

Relationship 2203 is considered applicable to all life-stages, as mitochondrial oxidative phosphorylation and ATP synthesis are essential energy production processes for maintaining basic biological activities.

Key Event Relationship Description

This key event relationship describes the dissipation of protonmotive force across the inner mitochondrial membrane by uncouplers (uncoupling of oxidative phosphorylation), leading to reduced total adenosine triphosphate (ATP) pool in cells or organisms.

Evidence Supporting this KER

The overall evidence supporting Relationship 2203 is considered high.

Biological Plausibility

The biological plausibility of Relationship 2203 is considered high.

Rationale: In eukaryotic cells, the major metabolic pathways responsible for ATP production are OXPHOS, citric acid (TCA) cycle, glycolysis and photosynthesis. Oxidative phosphorylation is much (theoretically 15-18 times) more efficient than the rest due to high energy derived from oxygen during aerobic respiration (Schmidt-Rohr 2020). As the ATP level is relatively balanced between production and consumption (Bonora 2012), ATP depletion is a plausible consequence of reduced ATP synthetic efficiency following uncoupling of OXPHOS.

Empirical Evidence

The empirical support of Relationship 2203 is considered high.

Rationale: The majority of relevant studies show good incidence, temporal and/or dose concordance in different organisms and cell types after exposure to known uncouplers, with relatively few exceptions.

Evidence:

- ***Temporal concordance***: Exposure of zebrafish embryos to 0.5 µM of the classical uncoupler 2,4-DNP led to significantly uncoupling of OXPHOS after 21h, whereas significant reduction in ATP was only observed after 45h (Bestman 2015).
- ***Dose concordance:*** The uncoupler triclosan induced significant uncoupling of OXPHOS in zebrafish embryos at 15 µM, whereas higher (30 µM) concentration was required to caused significant ATP depletion (Shim 2016).
- ***Dose concordance:*** Exposure to 1 µM of of the uncoupler CCCP led to 40% uncoupling of OXPHOS in rat RBL-2H3 cells, whereas the same magnitude of effect required 1.6 µM of CCCP (Weatherly 2016).
- ***Dose concordance:*** Exposure to 10 µM of the uncoupler triclosan caused significant uncoupling of OXPHOS in rat RBL-2H3 cells, whereas significant reduction in ATP was observed at a higher concentration (30 µM) (Weatherly 2018).
- ***Dose concordance:*** Significant effect on uncoupling of OXPHOS required 2 µM FCCP, whereas a significant reduction in required 20 µM FCCP in human RD cells (Kuruvilla 2003).
- ***Incidence concordance***: In human colon cancer cells (SW480), exposure to 150 µM of the uncoupler flavanoid morin caused 60% reduction in MMP, whereas only around 35% decrease in ATP (Sithara 2017).
- ***Incidence concordance:*** Exposure of rat RBL-2H3 cells to 10 µM of the uncoupler triclosan led to 50% uncoupling of OXPHOS, whereas only 40% reduction in ATP (Weatherly 2016).
- ***Incidence concordance:*** Exposure to 5 µM of the uncoupler CCCP caused 71% uncoupling of OXPHOS, whereas only 64% reduction of ATP in human HL-60 cells (Sweet 1999).
- ***Incidence concordance:*** Exposure of human HeLa cells to 50 µM of the uncoupler CCCP for 1h led to 77% uncoupling of OXPHOS and 25% reduction in ATP (Koczor 2009).
- ***Incidence concordance***: Exposure of the nematode Caenorhabditis elegans to 50 µM Arsenite for 1h led to approximately 45% uncoupling of OXPHOS and 20% reduction in ATP (Luz 2016).

Uncertainties and Inconsistencies

- A significant decrease followed by a significant increase in total ATP was observed in human RD cells during a 48h exposure to the uncoupler FCCP (Kuruvilla 2003), possibly due to the enhancement of other ATP synthetic pathways (e.g., glycolysis) as a compensatory action to impaired OXPHOS (Jose 2011

Quantitative Understanding of the Linkage

The quantitative understanding of Relationship 2203 is high.

Rationale: Multiple mathematical models have been developed for describing the quantitative relationships between uncoupling of OXPHOS and ATP synthesis in vertebrates (Beard 2005; Schmitz 2011; Heiske 2017; Kubo 2020). These models, however, are highly complex metabolic or systems biological models and warrant further simplification to be used for this AOP.

Response-response relationship

A regression based quantitative response-response relationship between uncoupling of OXPHOS and ATP depletion was proposed for the crustacean *Daphnia magna* under UVB stress (Song 2020).

Known Feedforward/Feedback loops influencing this KER

- It is known that mild uncoupling of oxidative phosphorylation can enhance the activity of the mitochondrial electron transport chain to produce more ATP, and/or activate other ATP synthetic pathways (e.g., glycolysis) as a compensatory action to impaired OXPHOS (Jose 2011).

References

Beard DA. 2005. A biophysical model of the mitochondrial respiratory system and oxidative phosphorylation. PLOS Computational Biology 1:e36. DOI: 10.1371/journal.pcbi.0010036.

Bestman JE, Stackley KD, Rahn JJ, Williamson TJ, Chan SS. 2015. The cellular and molecular progression of mitochondrial dysfunction induced by 2,4-dinitrophenol in developing zebrafish embryos. Differentiation 89:51-69. DOI: 10.1016/j.diff.2015.01.001.

Bonora M, Patergnani S, Rimessi A, De Marchi E, Suski JM, Bononi A, Giorgi C, Marchi S, Missiroli S, Poletti F, Wieckowski MR, Pinton P. 2012. ATP synthesis and storage. Purinergic Signalling 8:343-357. DOI: 10.1007/s11302-012-9305-8.

Heiske M, Letellier T, Klipp E. 2017. Comprehensive mathematical model of oxidative phosphorylation valid for physiological and pathological conditions. The FEBS Journal 284:2802-2828. DOI: https://doi.org/10.1111/febs.14151.

Jose C, Bellance N, Rossignol R. 2011. Choosing between glycolysis and oxidative phosphorylation: A tumor's dilemma? Biochimica et Biophysica Acta (BBA) - Bioenergetics 1807:552-561. DOI: https://doi.org/10.1016/j.bbabio.2010.10.012.

Koczor CA, Shokolenko IN, Boyd AK, Balk SP, Wilson GL, Ledoux SP. 2009. Mitochondrial DNA damage initiates a cell cycle arrest by a Chk2-associated mechanism in mammalian cells. J Biol Chem 284:36191-36201. DOI: 10.1074/jbc.M109.036020.

Kubo S, Niina T, Takada S. 2020. Molecular dynamics simulation of proton-transfer coupled rotations in ATP synthase FO motor. Scientific Reports 10:8225. DOI: 10.1038/s41598-020-65004-1.

Kuruvilla S, Qualls CW, Jr., Tyler RD, Witherspoon SM, Benavides GR, Yoon LW, Dold K, Brown RH, Sangiah S, Morgan KT. 2003. Effects of minimally toxic levels of carbonyl cyanide P-(trifluoromethoxy) phenylhydrazone (FCCP), elucidated through differential gene expression with biochemical and morphological correlations. Toxicol Sci 73:348-361. DOI: 10.1093/toxsci/kfg084.

Luz AT, Godebo TR, Bhatt DP, Ilkayeva OR, Maurer LL, Hirschey MD, Meyer JN. 2016. Arsenite Uncouples Mitochondrial Respiration and Induces a Warburg-Like Effect in Caenorhabditis elegans. Toxicol Sci 154:195-195. DOI: 10.1093/toxsci/kfw185.

Schmidt-Rohr K. 2020. Oxygen is the high-energy molecule powering complex multicellular life: fundamental corrections to traditional bioenergetics. ACS Omega 5:2221-2233. DOI: 10.1021/acsomega.9b03352.

Schmitz JPJ, Vanlier J, van Riel NAW, Jeneson JAL. 2011. Computational modeling of mitochondrial energy transduction. 39:363-377. DOI: 10.1615/CritRevBiomedEng.v39.i5.20.

Shim J, Weatherly LM, Luc RH, Dorman MT, Neilson A, Ng R, Kim CH, Millard PJ, Gosse JA. 2016. Triclosan is a mitochondrial uncoupler in live zebrafish. J Appl Toxicol 36:1662-1667. DOI: 10.1002/jat.3311.

Sithara T, Arun KB, Syama HP, Reshmitha TR, Nisha P. 2017. Morin inhibits proliferation of SW480 colorectal cancer cells by inducing apoptosis mediated by reactive oxygen species formation and uncoupling of Warburg effect. Frontiers in Pharmacology 8. DOI: 10.3389/fphar.2017.00640.

Song Y, Xie L, Lee Y, Tollefsen KE. 2020. De novo development of a quantitative adverse outcome pathway (qAOP) network for ultraviolet B (UVB) radiation using targeted laboratory tests and automated data mining. Environmental Science & Technology 54:13147-13156. DOI: 10.1021/acs.est.0c03794.

Sweet S, Singh G. 1999. Changes in mitochondrial mass, membrane potential, and cellular adenosine triphosphate content during the cell cycle of human leukemic (HL-60) cells. Journal of Cellular Physiology 180:91-96. DOI: https://doi.org/10.1002/(SICI)1097-4652(199907)180:1<91::AID-JCP10>3.0.CO;2-6.

Weatherly LM, Nelson AJ, Shim J, Riitano AM, Gerson ED, Hart AJ, de Juan-Sanz J, Ryan TA, Sher R, Hess ST, Gosse JA. 2018. Antimicrobial agent triclosan disrupts mitochondrial structure, revealed by super-resolution microscopy, and inhibits mast cell signaling via calcium modulation. Toxicol Appl Pharmacol 349:39-54. DOI: 10.1016/j.taap.2018.04.005.

Weatherly LM, Shim J, Hashmi HN, Kennedy RH, Hess ST, Gosse JA. 2016. Antimicrobial agent triclosan is a proton ionophore uncoupler of mitochondria in living rat and human mast cells and in primary human keratinocytes. Journal of Applied Toxicology 36:777-789. DOI: https://doi.org/10.1002/jat.3209.

Relationship: 2204: Decrease, ATP pool leads to Decrease, Cell proliferation

AOPs Referencing Relationship

AOP Name	Adjacency	Weight of Evidence	Quantitative Understanding

AOP Name	Adjacency	Weight of Evidence	Quantitative Understanding
Uncoupling of oxidative phosphorylation leading to growth inhibition via decreased cell proliferation	adjacent	Moderate	Moderate
Mitochondrial ATP synthase antagonism leading to growth inhibition (1)	adjacent		
Mitochondrial complex III antagonism leading to growth inhibition (1)	adjacent		

Evidence Supporting Applicability of this Relationship

Taxonomic Applicability

Term	Scientific Term	Evidence	Links
zebrafish	Danio rerio	High	NCBI
human	Homo sapiens	High	NCBI

Life Stage Applicability

Life Stage	Evidence
Embryo	High

Sex Applicability

Sex	Evidence
Unspecific	High

Taxonomic applicability

Relationship 2204 is considered applicable to all eukaryotes, as ATP and cell proliferation are known to be tightly coupled in animals, plants and some microorganisms.

Sex applicability

Relationship 2204 is considered applicable to all sexes, as ATP-dependent cell proliferation are used by both males and females in eukaryotes.

Life-stage applicability

Relationship 2204 is considered applicable to all life stages, as ATP-dependent cell proliferation is an essential process for an organism throughout the entire life.

Key Event Relationship Description

This key event relationship describes reduced adenosine triphosphate (ATP) supply leading to reduced cell proliferation (cell growth, division or a combination of these).

Evidence Supporting this KER

The overall evidence supporting Relationship 2204 is considered moderate.

Biological Plausibility

The biological plausibility of Relationship 2204 is considered high.

Rationale: Cell proliferation is a well-known ATP-dependent process. Cell division processes, such as the mitotic cell cycle uses ATP for chromosome movements and DNA replication (Kingston 1999). The synthetic processes of major cellular components that are necessary for cell structure and growth, such as proteins and lipids, also require sufficient ATP supply (Bonora 2012). Depletion of ATP therefore has a negative impact on these processes.

Empirical Evidence

The empirical support of Relationship 2204 is considered moderate.

Evidence:

- ***Incidence concordance***: Exposure of human HeLa cells to 50 µM of the uncoupler CCCP for 1h led to 25% reduction in ATP, whereas a non-significant reduction in cell proliferation (Koczor 2009).

- I*ncidence concordance*: Exposure of human RD cells to 20 µM of the uncoupler CCCP for 2h led to 20% ATP depletion, whereas a non-significant decrease in cell proliferation (Kuruvilla 2003).
- ***Incidence concordance***: Exposure of human SE480 cells to 150 µM of the uncoupler flavanoid morin for 48h led to 35% ATP depletion and 35% reduction in cell proliferation (Sithara 2017).

Uncertainties and Inconsistencies

There are currently no inconsistencies based on the supporting literature.

References

Ahmann FR, Garewal HS, Schifman R, Celniker A, Rodney S. 1987. Intracellular adenosine triphosphate as a measure of human tumor cell viability and drug modulated growth. In Vitro Cellular & Developmental Biology 23:474-480. DOI: 10.1007/BF02628417.

Bonora M, Patergnani S, Rimessi A, De Marchi E, Suski JM, Bononi A, Giorgi C, Marchi S, Missiroli S, Poletti F, Wieckowski MR, Pinton P. 2012. ATP synthesis and storage. Purinergic Signalling 8:343-357. DOI: 10.1007/s11302-012-9305-8.

Crouch SPM, Kozlowski R, Slater KJ, Fletcher J. 1993. The use of ATP bioluminescence as a measure of cell proliferation and cytotoxicity. Journal of Immunological Methods 160:81-88. DOI: https://doi.org/10.1016/0022-1759(93)90011-U.

Kingston RE, Narlikar GJ. 1999. ATP-dependent remodeling and acetylation as regulators of chromatin fluidity. Genes Dev 13:2339-2352. DOI: 10.1101/gad.13.18.2339.

Koczor CA, Shokolenko IN, Boyd AK, Balk SP, Wilson GL, Ledoux SP. 2009. Mitochondrial DNA damage initiates a cell cycle arrest by a Chk2-associated mechanism in mammalian cells. J Biol Chem 284:36191-36201. DOI: 10.1074/jbc.M109.036020.

Kuruvilla S, Qualls CW, Jr., Tyler RD, Witherspoon SM, Benavides GR, Yoon LW, Dold K, Brown RH, Sangiah S, Morgan KT. 2003. Effects of minimally toxic levels of carbonyl cyanide P-(trifluoromethoxy) phenylhydrazone (FCCP), elucidated through differential gene expression with biochemical and morphological correlations. Toxicol Sci 73:348-361. DOI: 10.1093/toxsci/kfg084.

Nieminen AL, Saylor AK, Herman B, Lemasters JJ. 1994. ATP depletion rather than mitochondrial depolarization mediates hepatocyte killing after metabolic inhibition. Am J Physiol 267:C67-74. DOI: 10.1152/ajpcell.1994.267.1.C67.

Sithara T, Arun KB, Syama HP, Reshmitha TR, Nisha P. 2017. Morin inhibits proliferation of SW480 colorectal cancer cells by inducing apoptosis mediated by reactive oxygen species formation and uncoupling of Warburg effect. Frontiers in Pharmacology 8. DOI: 10.3389/fphar.2017.00640.

Relationship: 2205: Decrease, Cell proliferation leads to Decrease, Growth

AOPs Referencing Relationship

AOP Name	Adjacency	Weight of Evidence	Quantitative Understanding
Uncoupling of oxidative phosphorylation leading to growth inhibition via decreased cell proliferation	adjacent	Moderate	Moderate
Mitochondrial ATP synthase antagonism leading to growth inhibition (1)	adjacent		
Mitochondrial complex III antagonism leading to growth inhibition (1)	adjacent		

Evidence Supporting Applicability of this Relationship

Taxonomic Applicability

Term	Scientific Term	Evidence	Links
zebrafish	Danio rerio	High	NCBI

Life Stage Applicability

Life Stage	Evidence
Embryo	High

Sex Applicability

Sex	Evidence
Unspecific	High

Taxonomic applicability

Relationship 2205 is considered applicable to all eukaryotes (both unicellular and multicellular), as growth (or population growth of alga) is well known to be achieved through cell proliferation in animals, plants and some microorganisms.

Sex applicability

Relationship 2205 is considered applicable to both all sexes, as cell proliferation leading to growth is a fundamental process and not sex-specific.

Life-stage applicability

Relationship 2205 is considered applicable to all life stages, as cell proliferation leading to growth is essential for maintaining basic biological processes throughout an organism's life.

Key Event Relationship Description

This key event relationship describes reduced cell proliferation (cell growth, division or a combination of these) leading to reduced tissue, organ or individual growth.

Evidence Supporting this KER

The overall evidence supporting Relationship 2205 is considered moderate.

Biological Plausibility

The biological plausibility of Relationship 2205 is considered high.

Rationale: The biological structural and functional relationship between cell proliferation and growth is well established. It is commonly accepted that the size of an organism, organ or tissue is dependent on the total number and volume of the cells it contains, and the amount of extracellular matrix and fluids (Conlon 1999). Impairment to cell proliferation can logically affect tissue and organismal growth.

Empirical Evidence

The empirical support of Relationship 2205 is considered low.

Rationale: Because cell proliferation is typically measured in vitro, while growth of an organism is measured in vivo, few studies have measured both in the same experiment. There is one zebrafish study reporting concordant relationship between reduced cell proliferation and embryo growth with some inconsistencies (Bestman 2015).

Uncertainties and Inconsistencies

- In zebrafish embryos exposed to 2,4-DNP, significant growth inhibition (AO) was identified after 21h, whereas a non-significant reduction in cell proliferation was observed (Bestman 2015).

References

Bestman JE, Stackley KD, Rahn JJ, Williamson TJ, Chan SS. 2015. The cellular and molecular progression of mitochondrial dysfunction induced by 2,4-dinitrophenol in developing zebrafish embryos. Differentiation 89:51-69. DOI: 10.1016/j.diff.2015.01.001.

Binder BJ, Landman KA, Simpson MJ, Mariani M, Newgreen DF. 2008. Modeling proliferative tissue growth: a general approach and an avian case study. Phys Rev E Stat Nonlin Soft Matter Phys 78:031912. DOI: 10.1103/PhysRevE.78.031912.

Conlon I, Raff M. 1999. Size control in animal development. Cell 96:235-244. DOI: 10.1016/s0092-8674(00)80563-2.

Jarrett AM, Lima EABF, Hormuth DA, McKenna MT, Feng X, Ekrut DA, Resende ACM, Brock A, Yankeelov TE. 2018. Mathematical models of tumor cell proliferation: A review of the literature. Expert Review of Anticancer Therapy 18:1271-1286. DOI: 10.1080/14737140.2018.1527689.

Mosca G, Adibi, M., Strauss, S., Runions, A., Sapala, A., Smith, R.S. 2018. Modeling Plant Tissue Growth and Cell Division. In Morris R., ed, Mathematical Modelling in Plant Biology. Springer, Cham.
